# Vemurafenib inhibits immune escape biomarker BCL2A1 by targeting PI3K/AKT signaling pathway to suppress breast cancer

**DOI:** 10.3389/fonc.2022.906197

**Published:** 2022-11-29

**Authors:** Yalan Dai, Liqiong Yang, Abass Sakandar, Duoli Zhang, Fukuan Du, Xinyi Zhang, Linglin Zou, Yueshui Zhao, Jigang Wang, Zhenhua Zhang, Xu Wu, Mingxing Li, Xiao Ling, Lei Yu, Lishu Dong, Jing Shen, Zhangang Xiao, Qinglian Wen

**Affiliations:** ^1^ Department of Oncology, Affiliated Hospital of Southwest Medical University, Luzhou, Sichuan Province, China; ^2^ Laboratory of Molecular Pharmacology, Department of Pharmacology, School of Pharmacy, Southwest Medical University, Luzhou, Sichuan, China; ^3^ Cell Therapy & Cell Drugs, Luzhou Key Laboratory, Luzhou, China; ^4^ South Sichuan Institute of Translational Medicine, Southwest Medical University, Luzhou, China; ^5^ School of Data Science, The Chinese University of Hong Kong, Shenzhen, China; ^6^ Shenzhen Municipal People’s Hospital, Shenzhen, China; ^7^ Department of Obstetrics, Luzhou Maternal & Child Health Hospital (Luzhou Second People’s Hospital), Luzhou, Sichuan, China

**Keywords:** Immune escape, breast cancer, prognostic model, bioinformatics, biomarker, BCL2A1, vemurafenib, PI3K/Akt signaling pathway

## Abstract

**Objectives:**

To investigate the role of immune escape encoding genes on the prognosis of BC, and to predict the novel targeting agents.

**Methods:**

Human immune genes and immune escape encoding genes were obtained from the IMMPORT database and the previous study. Sample information and clinical data on BC were obtained from the TCGA and GTEX databases. Obtaining differentially expressed protein data from cBioportal database. To construct a risk score model by lasso analysis, and nomogram was used to predict score core. GSCA, TIMER and CELLMINER databases were used for immune and drug susceptibility correlation analyses. Cell experiments were verified by MTT, Western blotting, and RT-qPCR.

**Results:**

We found prognostic models consisting of eleven immune escape related protein-coding genes with ROC curves that performed well in the ontology data (AUC for TCGA is 0.672) and the external data (AUC for GSE20685 is 0.663 and for GES42568 is 0.706). Five core prognostic models are related to survival (EIF4EBP1, BCL2A1, NDRG1, ERRFI1 and BRD4) were summarized, and a nomogram was constructed to validate a C-index of 0.695, which was superior to other prognostic models. Relevant drugs targeting core genes were identified based on drug sensitivity analysis, and found that Vemurafenib downregulates the PI3K-AKT pathway and BCL2A1 protein in BC, as confirmed by external data and cellular assays.

**Conclusions:**

Briefly, our work establishes and validates an 11-immune escape risk model, and five core prognostic factors that are mined deeply from this model, and elucidates in detail that Vemurafenib suppresses breast cancer by targeting the PI3K/AKT signaling pathway to inhibit the immune escape biomarker BCL2A1, confirms the validity of the prognostic model, and provides corresponding targeted agents to guide individualized treatment of BC patients.

## Introduction

The World Health Organization’s International Agency for Research on Cancer (IARC) released the latest data on the global cancer burden in 2020. New cases of breast cancer (BC) increased rapidly to 2.26 million, accounting for 11.7% of all new cancer patients, officially overtaking lung cancer (2.2 million cases) as the world’s leading cancer for the first time (1). Effective prevention, diagnosis, treatment, and whole-process management strategies for BC have become important mediums for improving the global cancer burden.

The efficacy of local and systemic treatment of BC has improved greatly in recent years, and avoid the underutilization of medical resources has become a focus (2). The clinical decision of BC is mainly dependent on the abnormal expression of estrogen, progesterone endocrine receptors (ER, PR) and HER2. Immunotherapy has been shown to have salvage implications in many cancers. Antibodies against the immunomodulators PD-L1/PD-1 and CTLA4 have had staged clinical success, but more in-depth studies of immunotherapy are still needed. Proper functioning of the immune system requires constant modulation to ensure protection against foreign factors and the tolerance of autoantigens (3).

Immune editing is a dynamic process that regulates tumor evolution through the immune system, including elimination, homeostasis and escape (4). Immune escape is key to the persistence of most solid tumors, and it is a key obstacle to the success of cancer (immune) treatment (5). The mechanisms of immune escape include a decrease in immune analysis, downregulation of co-stimulatory molecules, and overexpression of co-inhibitory molecules, causing a decrease in CD8+ T cell activity and weakening the body’s anti-tumor immunity (6). Immune escape is a necessary condition for the formation and development of breast tumors and a key step in the transition from pre-invasive lesions to aggressive tumors (7).

Current work is focused on developing combination therapies to convert non-responders into responders, deepen existing responses, and overcome acquired immunotherapy resistance (8). The response elicited by immunotherapy is expected to clearly target and destroy tumor cells while preserving normal cells. Immunotherapeutic approaches include the use of antibodies to neutralize or block immune checkpoints, induction of proliferation and/or increased activity of tumor infiltrating lymphocytes (CTL), and it regulating the tumor microenvironment (TME) (9). Effective anti-tumor strategies must focus on targeting multiple immune pathways to fully activate endogenous tumor immunity (10, 11). In advanced BC patients, immunotherapy combined with targeted therapy is undergoing basic and clinical trials to confirm biomarkers in the tumor and TME, as well as to identify the pharmacokinetics and pharmacodynamics of drug combinations, and to optimize drug dosing in order to find the optimal combination of treatments for individual patients (12).

BC is extremely heterogeneous at the clinical and molecular level. Because of the use of various histological techniques(genomics, transcriptomics or proteomics, etc.), we have gained deep understanding of the complexity of the development of BC (13). Multiomic is an emerging analytical approach that combines next-generation DNA and RNA sequencing with protein characterization to provide features such as protein expression levels, post-translational modifications, and protein-protein interactions (14).

Keith A. Lawson et al. have found a key set of genes and pathways, which make tumor cells dodge CTL-mediated killing. They found 182 immune escape related genes by the genome wide CRISPR screen on a set of mouse BC cell lines cultured in the presence of CTL, and disrupting these genes alone increased the sensitivity and resistance of cancer cells to CTL killing (15). There are no comprehensive studies on the genes encoding immune escape in BC, and endocrine therapy plus CDK4/6 inhibitors have become the standard of care for estrogen receptor-positive (ER+) BC. Although immune checkpoint inhibitors (ICIs) have shown promising antitumor activity in a variety of cancer types, only limited success has been achieved in patients with metastatic breast cancer (mBC), particularly the ER+ subtype, with typically exhibiting a lower tumor mutational load (TMB) compared to other subtypes and are therefore considered immunostasis. This may lead to missed treatment opportunities and overdosing (16, 17).

Targeted therapies have been greatly developed in BC, such as Toremifene and Pertuzumab (18). Vemurafenib is often used in BRAF-mutated melanoma and is gradually being explored in BC. A xenograft model of vemurafenib-treated MDA-MB-231 showed growth-inhibitory activity associated with inhibition of tumor angiogenesis (19). Magdalena Pircher et al. reported the first successful control of multiple lung metastases from triple-negative BC with vemurafenib (20). The specific mechanism by which vemurafenib inhibits breast cancer needs to be further explored.

The main objective of this study is to screen for immune escape related genes, and to identify hub genes that are associated with prognosis and efficacy of BC, and to provide new targets for the treatment of BC.

## Materials and methods

### Data

182 core immune escape encoding genes were extracted from a study by Keith A. Lawson. et al. IMMPORT database (https://www.immport.org/) provided 2483 immune encoding genes (21). Venn diagram was drawn by Hiplot mapping tool (https://hiplot.com.cn/) to identify two sets of intersection genes. Downloaded data from TCGA database (https://tcga-Data.Nci.nih.gov/TCGA/), GTEX database (https://www.Gtex-portal.org/home/) and UCSC database (http://xena.Ucsc.edu/), which provided us with clinical data from 1095 BC cases and 292 normal controls, and their matched transcriptome RNA second-generation sequencing data.

### Differentially expressed protein analysis and enrichment analysis

The “t-test” of “GraphPad Prism 8” were used for differential expression analysis of normal and tumor samples, and the “survival” R package for survival analysis. The cBioportal database (http://www.cbioportal.org/) was used for further differentially expressed proteins analysis (22). Gene ontology (GO) and Kyoto Encyclopedia of Genes and Genomes (KEGG) enrichment analyses of differential expression of protein-coding genes were performed using “clusterprofiler” package. Category, Count, and -log10pvalue were used by GO analysis to obtain important metabolic pathways.

### Cox regression analysis to establish a risk model

First, the gene sequencing data and patient survival data were combined, and univariate Cox analysis was performed using the “surviving” and “forestplot” R packages, and critical genes associated with prognosis were further explored using the “glmnet” R package. Multivariate cox analysis of candidate genes was performed with the “surv” package, and it constructed a prognostic risk assessment model.


$$Risk\;score=\sum_{i=0}^{n}\left(Expi*Coei\right)$$

Then, risk prediction and risk score calculation were performed using a prediction model. Based on the risk scores, patients were divided into high- and low- risk groups.

### Measure the risk model

The “Hiplot” plotting tool was used to show the distribution of survival situation of low- and high- risk patients. The “pheatmap” R package was used to observe the difference in expression levels between high- and low- risk groups. Kaplan-Meier Survival analysis was used to assess the survival rates of BC patients in both groups. The validity of candidate genes in predicting the prognosis of BC patients was measured by plotting ROC curves using the “pROC” R package, which based on survival status, survival time and candidate gene expression in BC patients. The TCIA database (https://tcia.at/home) was used to analysis the enrichment of immune cells in the high- and low- risk groups (23). The prognostic model was externally validated by GSE20685 and GES42568 datasets.

### Screening of core prognostic factors and construction of nomogram

The “T test” of “GraphPad Prism 8” was used for differential expression analysis of normal and tumor samples, and “survival” package was used to obtain survive-related hub genes, and P<0.05 was regarded statistically significant. The nomogram was used to predict the effect of each differentially expressed protein coding genes on 1-, 3-, and 5- year overall survival. The nomogram is composed of central prognostic factors, whose point scale is assigned to each variable. We used a horizontal line to determine the points for each variable and calculated the total points for each patient by adding points for all variables by normalizing the distribution from 0 to 100. Then we established the performance of the calibration curve for the visual nomogram. We compared the predictions in the calibration curves with the observed results. The best prediction was when the slope was close to 1.

### Immunoassay database

The “XY” of “GraphPad Prism 8” performed correlation analysis of central protein-encoding genes with immune checkpoints PD-1, PD-L1 and CTLA4 and showed the relationship between them using Hiplot’s chord charts. The Gene Set Cancer Analysis (GSCA) database (http://bioinfo.life.hust.edu.cn/GSCA/) was used to analyze the expression of central protein-encoding genes and associated immune infiltration (24). The TIMER database (https://cistrome.shinyapps.io/timer/) was used to capture the immune cells and the correlation of tumor purity and quantity of gene expression, and represented by the heat map of the Hiplot (25).

### Drug sensitivity analysis database

Used the CELLMINER database (https://discover.nci.nih.gov/cellminer/) download the processed data “Processed Data Set” (26), and the data was processed by the “readxl” package. There were some “NA” missing values in the drug sensitivity data, and the “impute. KNN ()” function was used to evaluate and complement the missing values. Pearson correlation coefficients between each gene expression and different drugs were calculated and the two groups of drugs with the maximum and minimum correlation for each protein-coding gene were found based on the correlation coefficients. Correlation analysis was performed using “XY” of “GraphPad Prism 8” for visualization and validation purposes. The GES97681 dataset was used to verify whether Vemurafenib affects the expression of B-cell lymphoma 2-associated protein A1 (BCL2A1) and to find the pathway of drug effects through the literature, integrating the genes and pathways in the KEGG data (https://www.genome.jp/kegg/) to discover the effects of drugs on genes.

### Cell culture

Human BC lines (MCF-7, MDA-MB-231, SK-BR-3) were obtained from the School of Biomedical Sciences, Chinese University of Hong Kong. MCF-7 maintained in RPMI-1640 medium and MDA-MB-231 were cultured in Dulbecco’s modified Eagle’s medium (DMEM) (Gibco; Thermo Fisher Scientific, Inc.). Both media contained 10% fetal bovine serum (FBS; Thermo Fisher Scientific, Inc.) and 100 μg/ml streptomycin/100 U/ml penicillin (Gibco; Thermo Fisher Scientific, Inc.). SK-BR-3 were cultured in DMEM complete medium (iCell-h189-001b). Human breast epithelial cells (MCF-10A) purchased from iCell Bioscience Inc, China. MCF-10A was cultured in special medium (iCell-h131-001b). Vemurafenib (MedChemExpress, HY-12057) was dissolved in dimethyl sulfoxide (DMSO, final concentration is 0.1%) to prepare required concentrations. All cell lines were kept at 37°C in a humidified incubator with 5% CO2.

### MTT assay

For the MTT assay, MCF-7, SK-BR-3 and MDA-MB-231 cells were detached by trypsinization and counted in a haemocytometer. Cells were seeded in 96-well plates at a density of 4000 cells/well in 100 μl medium per well. Twenty-four hours after incubation in the CO2 incubator, adherent cells were treated with increasing concentrations of drugs: vemurafenib (0, 20, 40, and 60 μM) in fresh 1640 and DMEM medium. For measuring the concentration of vemurafenib that resulted in 50% control growth inhibition (IC50), at 48 hours following drug treatment, 10 μl MTT (3-(4,5-Dimethylthiazol-2-yl)-2,5-diphenyltetrazolium bromide) (Sigma-Aldrich) solution (5 mg/ml in PBS) was added to each well after 48h of drug treatment. In addition, cell viability was measured by adding MTT at 0, 24, 48, 72h. Then incubation was continued for additional 4h in the CO2 incubator. The blue formazan product, formed by reduction in live attached cells, was dissolved by adding 100 μl of 100% DMSO per well. The plates were gently swirled at room temperature for 10 minutes to dissolve the precipitate. Absorbance was monitored at 490 nm using a microplate reader (CYTATION 3; Agilent Technologies, Inc.). The effect of vemurafenib on cell viability was assessed as the percent of cell viability compared with vehicle-treated control cells, which were arbitrarily assigned 100% viability. Dose-response curves and IC50 values were obtained using GraphPad Prism software. At least 3 dose-response experiments were performed for each compound, and the mean IC50 ± SD was calculated.

### Real-Time Quantitative Polymerase Chain Reaction (RT-qPCR)

Cells were seeded at the density described above and incubated for 24 hours before the addition of vemurafenib. The compounds were added to the final concentrations of 0, 20, 40, and 60 μM. After 48 hours of treatment, Cell pellets were collected for experiments. Total RNA was isolated from MCF-7, SK-BR-3 and MDA-MB-231 by using Trizol reagent (Thermo Fisher Scientific, Inc.) according to the manufacturer’s instructions. Total RNA (1 μg) from each group of treated cells was converted to cDNA using a FastKing RT reagent kit (Tiangen, Inc.). The RT reaction was performed at 42°C for 15 min and 95°C for 3 min. qPCR was performed with a SYBR Green Real Time PCR kit (Thermo Fisher Scientific, Inc.) on CFX96 Touch Real Time PCR System (BioRad Laboratories, Inc.) under the following conditions: 95°C for 1 min, then 40 cycles of 95°C for 5 sec and 60°C for 15 sec. The gene expression level was calculated as 2(^−△△Ct^) method, and the △Ct means the Ct of target gene minus the Ct of reference gene. The primers used for real-time PCR were BCL2A1 (forward) 5’-AAATTGCCCCGGATGTGGAT-3’ and (reverse) 5’-ACAAAGCCATTTTCCCAGCCT-3’; GAPDH (forward) 5’-CTGGGCTACACTGAGCACC-3’ and (reverse) 5’-AAGTGGTCGTTGAGGGCAATG-3’.

### Western blot analysis

Western blotting was used to test the PI3K/AKT signaling pathway and BCL2A1 protein. RIPA lysis buffer (Beijing Solarbio Science & Technology Co., Ltd.) containing protease inhibitor PhosSTOP EASYpack (Roche Diagnostics) was used for total protein extraction according to the manufacturer’s protocol. Protein concentration was measured by the BCA kit (Beyotime Biotechnology, China). Then, protein samples were separated by 10% SDS−PAGE gel and transformed into PVDF membranes (Millipore, USA). Afterwards, membranes were incubated using 5% bovine serum albumin (BSA, Sigma−Aldrich; Merck KGaA) at room temperature for 1h and incubated with primary antibodies under 4°C overnight. The antibodies are as follows: anti-PI3K (1: 1,000, 4249, CST), anti-AKT (1: 1,000, 9272S, CST), anti-phosphorylated (p)−AKT (1: 1,000, 9271S, CST), anti-BCL2A1 (1: 1,000, 14093, CST), and anti-GAPDH (1: 2,000, GTX100118, GENE TEX) with GAPDH being the endogenous control. Afterwards, membranes were incubated with HRP−conjugated secondary antibodies at room temperature for 1h using a secondary antibody (1: 3,000, A0208, Beyotime). Finally, ECL blotting detection reagents (Clarity; Bio−Rad Laboratories, Inc.) was utilized to observe protein blots and the signals were detected by the ChemiDoc™ Imaging System (Bio−Rad Laboratories, Inc.).

### Datasource

Databases we used have: IMMPORT database, TCGA database, GTEX database, UCSC database, cBioportal database, TCIA database, GEO database (include GSE20685, GES42568 and GES97681 datasets), GSCA database, TIMER database, CELLMINER database. The softwares we used have: Hiplot mapping tool and GraphPad Prism.

### Statistical analysis

Our research mainly used “GraphPad Prism 8” and “R language” for data difference analysis, visualization, etc. From the “GraphPad Prism 8”, “T test” was used to compare the differences between two data sets, “one-way ANOVA” was used to compare differences between three or more groups of data, and “XY” was used for correlation analysis. P<0.05 was considered statistically significant, indicating significant differences between the data were used. R is the language and operating environment for statistical analysis and mapping. we used the “Pheatmap”, “Survival”, “GGPUBR” and “clusterprofiler “ package fort expression analysis, survival analysis and enrichment analysis for differentially expressed genes and proteins. All experiment data are presented as the mean ± standard deviation (SD), further analyzing using GraphPad Prism 9.0. Differences in the results of two groups were evaluated using either two-tailed Student’s t test or one-way ANOVA followed by *post-hoc* Dunnett’s test. The differences with P< 0.05 were considered statistically significant.

## Results

### Fifteen immune escape encoding genes significantly associated with the survival and prognosis of BC

**Figure 1:**
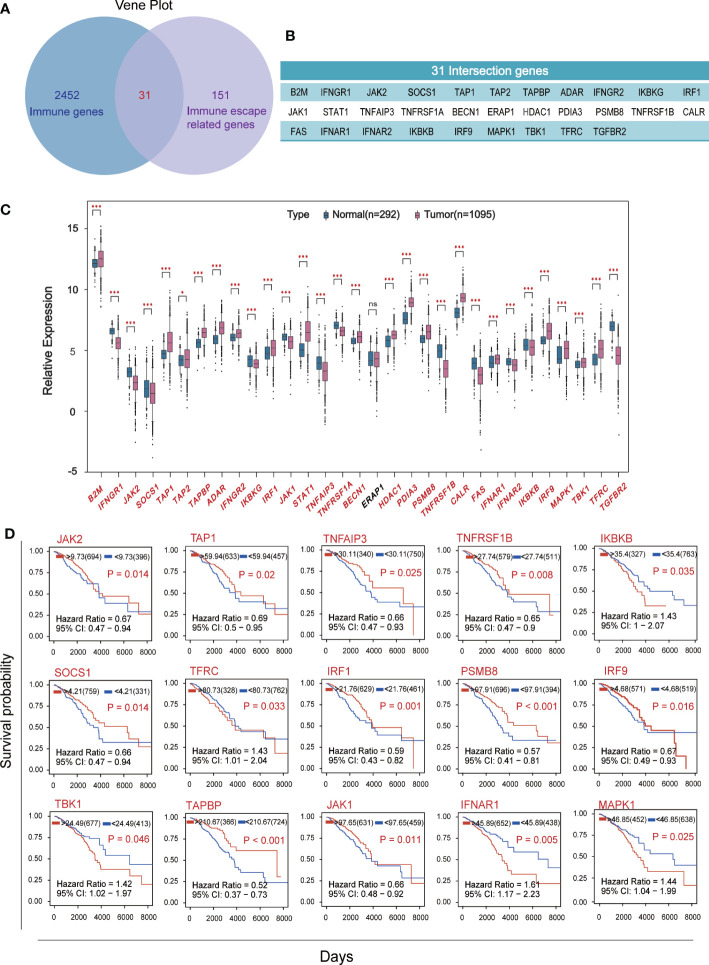
Screening for hub genes associated with immune escape from breast cancer. **(A)** The overlap of 2483 immune-related genes from Immport and 182 immune-escape related genes. **(B)** Display of 31 intersection genes. **(C)** The expression levels of breast cancer immune escape genes were analyzed using RNA sequencing data from 1095 cancer patients and 292 healthy subjects in the TCGA and GTEX databases. **(D)** Kaplan-Meier survival curve of immune escape genes in breast cancer based on expression level. Fifteen hub genes which P<0.05 and with significant differences in expression levels were shown. (★p<0. 05,★★p<0. 01 and★★★p< 0.001).

Venn plot was used to identify the overlap of 2483 human immune associated genes from Immport database with 182 immune escape associated genes from Keith A. Lawson’s study (**Figure 1A**), yielded 31 crossover genes (**Figure 1B**). We compared the expression levels of immune escape encoding genes between BC patients and healthy individuals, found that 18 genes were significantly up-regulated and 12 genes were significantly down-regulated in BC patients, and ERAP1 was not significantly different in BC patients and healthy individuals (**Figure 1C**). BC patients were divided into high and low groups according to “res.cut” expression levels, and Kaplan-Meier survival analysis was performed for BC immune escape gene expression levels. Among them, 15 genes showed significant differences in survival and expression levels in BC patients (**Figure 1D**), and the survival curves of the other 16 genes were also shown (**Figure S1A**).

### Immune escape in BC is mainly influenced by PI3K-Akt signaling pathway

**Figure 2:**
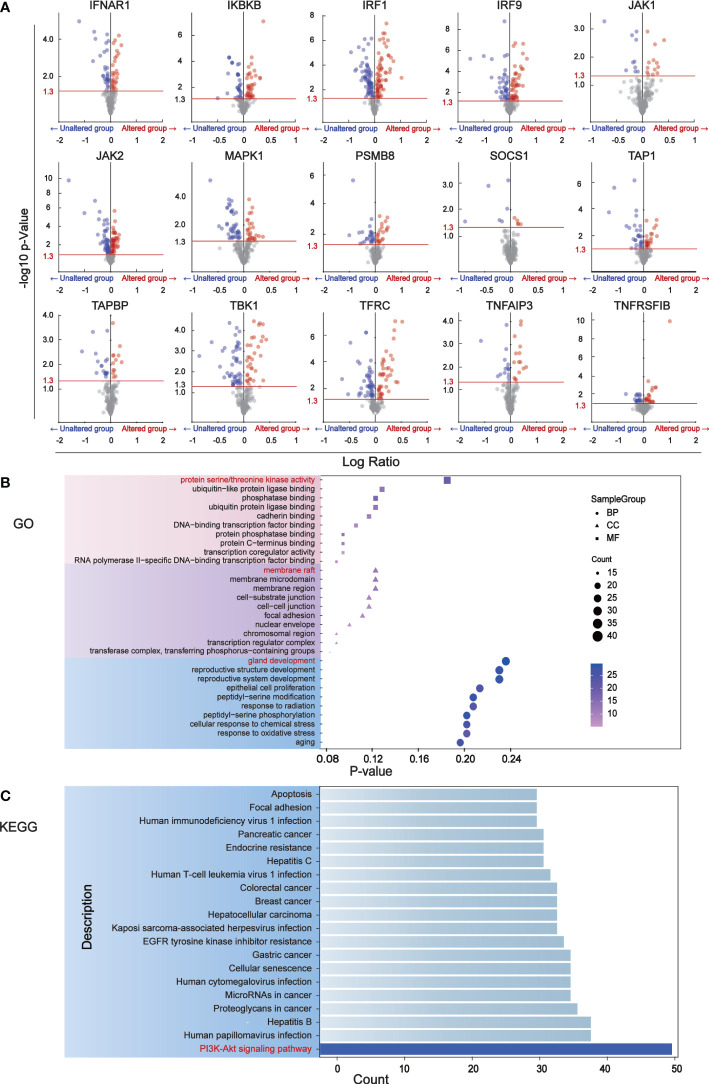
The major signaling pathway affected by genes associated with immune escape. **(A)** Volcano mapping to identify differential expressed proteins affected by immune escape related genes and analyzed by reverse phase protein array (RPPA) in cBioPortal. The Y-axis is the value that expresses the change in the horizontal fold, based on the logarithmic ratio (average of the changing expression/average of the constant expression). -log10 (p value)>1.30 is considered a significant difference. **(B)** The bubble diagram of GO enrichment analysis of immune escape gene was analyzed through clusterProfiler. Ten proteins have been shown to be important in biological processes, cell components, and molecular functions. **(C)** The downstream pathways related to immune escape gene changes were analyzed by KEGG pathway in clusterProfiler.

We identified 181 differentially expressed proteins regulated by BC immune escape encoding genes through the cBioPortal database (**Table S1**). The volcanic plots (**Figure 2A**) show proteins with significant changes (P<0.05) and were used for further analysis. To determine which pathway differential proteins were mainly enriched, we performed GO and KEGG enrichment analysis using “clusterprofiler” R package, which showed that these differentially expressed proteins play important roles in protein serine/threonine kinase activity, ubiquitin-like protein ligase binding, membrane raft, cell substrate connection, gland development andreproductive structure development. The PI3K-Akt signaling pathway is considered to be the most prominent downstream signaling pathway for immune escape related genes in BC (**Figures 2B, C**).

### An 11-gene prognostic risk model was constructed and validated with external data sets

**Figure 3:**
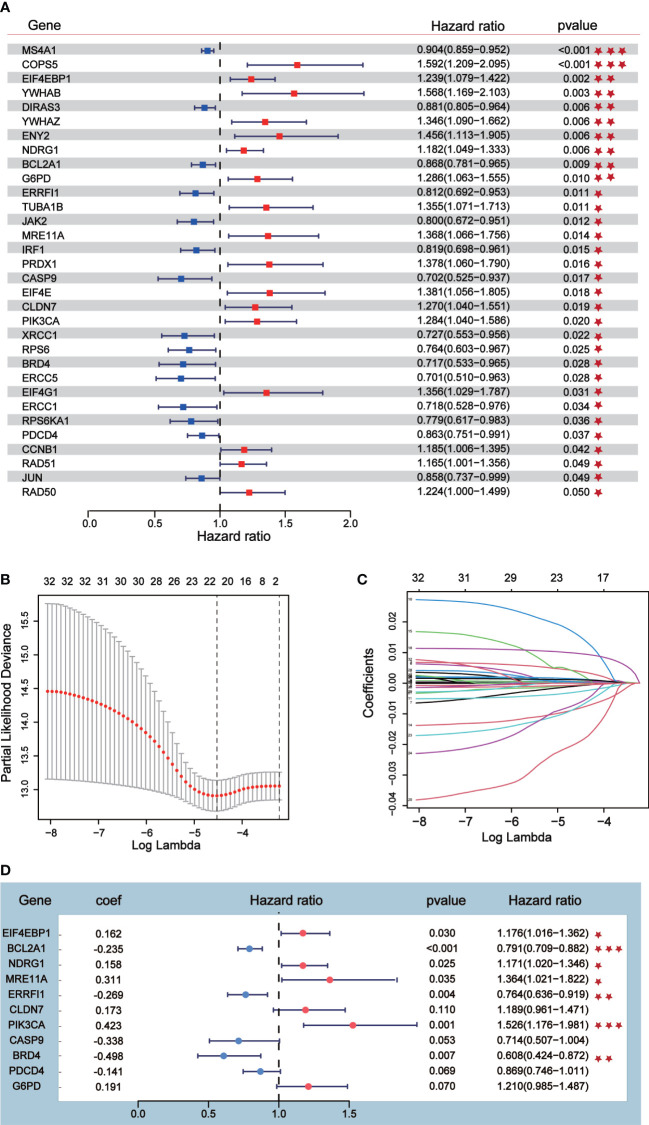
Prognostic risk model construction. **(A)** Univariate Cox analysis identified 31 differential expressed proteins coding genes related to survival. **(B, C)** Sixteen differential expressed proteins coding genes were further screened out from 31 genes by Lasso regression analysis and tenfold cross validation. **(D)** Multivariate Cox analysis was conducted according to Lasso results to obtain a risk model.

**Figure 4:**
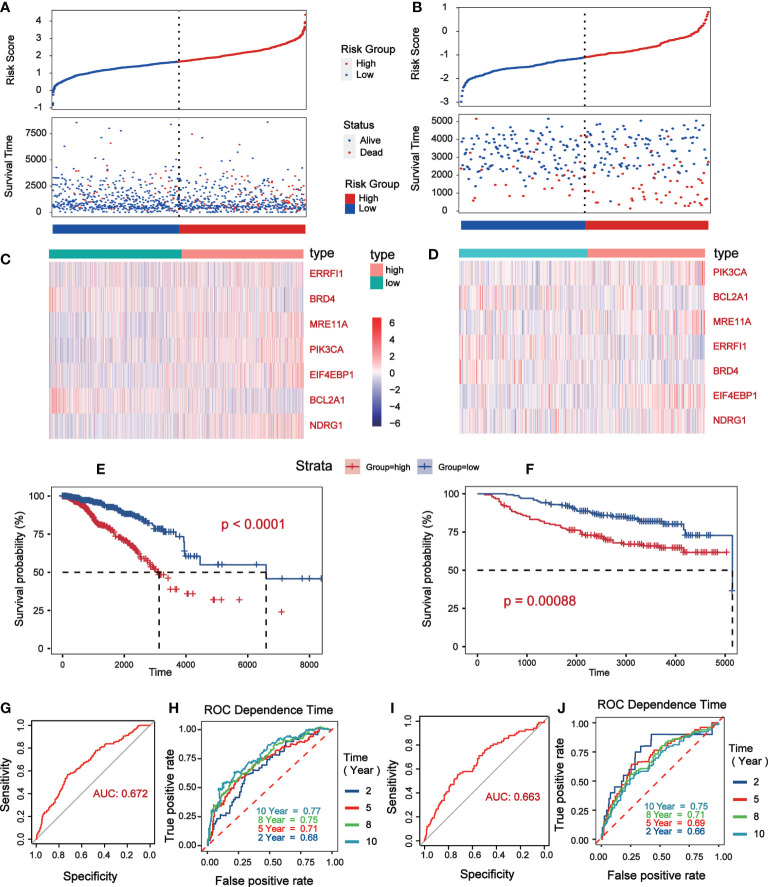
Verification of prognostic risk model based on TCGA database (Data were collected from low risk groups (n = 564) and high risk groups (n = 504)) and GSE20685 (Data were collected from low risk groups (n = 171) and high risk groups (n = 156)). **(A, B)** The status distribution of patients’ survival between low and high risk groups. **(C, D)** Heat map showing expression levels of high and low genetic risk groups. **(E, F)** Kaplan–Meier curves of OS between the high risk and low risk groups. **(G, H)** The P-ROC curve verified the risk model, and the AUC>0.65 was considered to have good predictive value. **(I, J)** The time of 2- year, 5- year, 8- year and 10 - year survival forecasts depends on the ROC curve.

Univariate Cox analysis was performed on 181 differentially expressed protein coding genes associated with immune escape in BC, and 31 of them were identified to be associated with survival (**Figure 3A**). To evaluate differentially expressed protein-coding genes meaningfully associated with BC prognosis and to obtain a better-fitting model, we used LASSO to downscale the high-dimensional information by adding a constraint to the absolute value of the coefficients and further screening by 10-fold cross-validation. The optimal λ value (λmin=0.025) was obtained from the minimum local likelihood deviation, and 16 genes were found to be significantly associated with prognosis (**Figures 3B, C**). The previous one looks at each feature individually to see if it is related to survival, while multivariate cox regression is to test whether multiple features are related to survival at the same time. Multivariate Cox analysis recognized 11 excellent prognostic gene models, namely EIF4EBP1, BCL2A1, NDRG1, MRE11A, ERRFI1, CLDN7, PIK3CA, CASP9, BRD4, PDCD4, and G6PD (**Figure 3D**). The risk score of patients was reckoned based on the scoring formula: Risk score = (EIF4EBP1 expression level * 0.162425485) - (BCL2A1 expression level * 0.234522698) + (NDRG1 expression level * 0.158199675) + (MRE11A expression level * 0.3105604) - (ERRFI1 expression level * 0.268609023)+ (CLDN7 expression level * 0.173133629) + (PIK3CA expression level * 0.442654646) - (CASP9 expression level * 0.337535821) - (BRD4 expression level * 0.497664344) - (PDCD4 expression level * 0.140816004) + (G6PD expression level * 0.190558316). There were more deaths in the high-risk group than the low-risk group, while fewer patients survived longer than 5 years than in the low-risk group (**Figure 4A**). The heatmap shows the risk of prognostic genes with a multivariate outcome P value less than 0.05. As the risk score increased, the expression levels of MRE11A, PIK3CA, EIF4EBP1 and NDRG1 increased, while the expression levels of BCL2A1 and BRD4 decreased (**Figure 4C**). Kaplan-Meier survival curve assessed the difference in survival between high- and low-risk patients in the risk model, which showed that survival decreased more significantly over time in the high-risk group than in the low-risk group, and the mean prognosis was worse in the high-risk group than in the low-risk group (P<0.0001) (**Figure 4E**). The prognostic value of Kaplan-Meier survival curves was determined by the P-ROC curve, and the area under the curve (AUC) was 0.672, indicating the median reliability of the kaplan-Meier survival curve (**Figure 4G**). The AUC of the survival assessment model was 0.68 for 2 years, 0.71 for 5 years, 0.75 for 8 years, and 0.77 for 10 years (**Figure 4I**). The applicability of the prognostic model was externally validated using the GSE20685 dataset (**Figures 4B, D, F, H, J**) and GES42568 dataset (**Figure S2**), and the results further confirmed the reliability of the risk model. We imported the high- and low- risk groups into the TCIA database and found that the high-risk groups had a lower percentage of immune cells clustering (**Figure S2**).

### Nomogram validates the predictive value of 5 core prognostic factors

**Figure 5:**
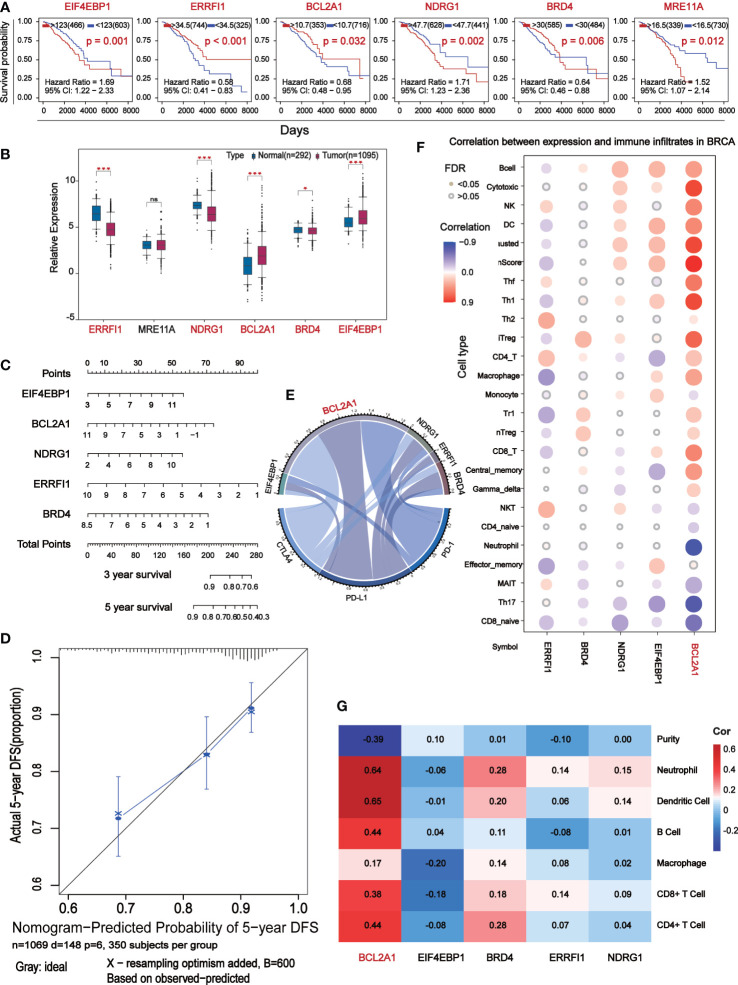
The central differential expressed proteins were further screened for immunocorrelation analysis. **(A)** Kaplan-Meier survival analysis based on the result of Multivariate Cox which P<0.05. **(B)** Box-plot analyze survival analysis for P<0.05 expression level of marker. **(C)** Prognostic maps of five central gene expression levels were established to predict overall survival at 1, 3, and 5 years. **(D)** Calibration chart of Nomogram. The black line represents the predicted results and the blue line represents the actual results. The high agreement between the two indicates that the prediction results are reliable. **(E)** Chord diagram shows the correlation between five central protein-coding genes and immune checkpoints. The longer arc and larger area with the better correlation. **(F)** Correlation analysis of the five central protein-coding genes and 26 immune cells: red represents positive correlation, blue represents negative correlation, the darker color show the stronger the correlation, and the small solid circles in color indicate FAD<0.05. **(G)** Correlation analysis (TIMER) between five central protein-coding genes and tumor purity and 6 immune cells.

Seven of the 11 prognostic indicators were statistically significant, when Kaplan-Meier survival analysis was performed on them according to expression level, six of them were significantly different (**Figure 5A**). Analysis of their expression levels in tumor patients and normal subjects identified five core prognostic markers, which were EIF4EBP1, BCL2A1, NDRG1, ERRFI1 and BRD4 (**Figure 5B**). We plotted the expression levels of five central prognostic factors based on 1069 TCGA-BC patients. By testing these parameters to obtain the corresponding assigned scores for their expression, the individual gene scores were summed to find the total score, which can predict the survival of BC patients for 1, 3, and 5 years (**Figure 5C**). A calibration curve was plotted to verify the accuracy of the prediction of the line graph. In the calibration diagram, the predicted result (blue line) was very close to the actual result (black line), with a c-index of 0.695, indicating that the prediction quality of Nomogram was very high (**Figure 5D**). In conclusion, five core prognostic markers can accurately predict the prognosis of BC patients.

### BCL2A1 is significantly associated with BC immunity

Correlation analysis of 5 core prognostic factors and immune checkpoints (PD-1, PD-L1 and CTLA4) showed that BCL2A1 had the highest correlation with immune checkpoints, while other factors with no significant difference (**Figure 5E**). Correlation analysis of the 5 central protein-encoding genes with 26 immune cells showed that BCL2A1 had the highest correlation with immune cells (**Figure 5F**). The correlation of 5 central protein-coding genes with tumor purity and 6 kinds of immune cells (B cells, CD4+ T cells, CD8+ T cells, Neutrphils, Macrophages and Dendritic cells) was further analyzed. BCL2A1 was negatively correlated with tumor purity and positively correlated with immune cells (**Figure 5G**).

### Analysis of the expression of five core prognostic factors in different BC subtypes

**Figure 6:**
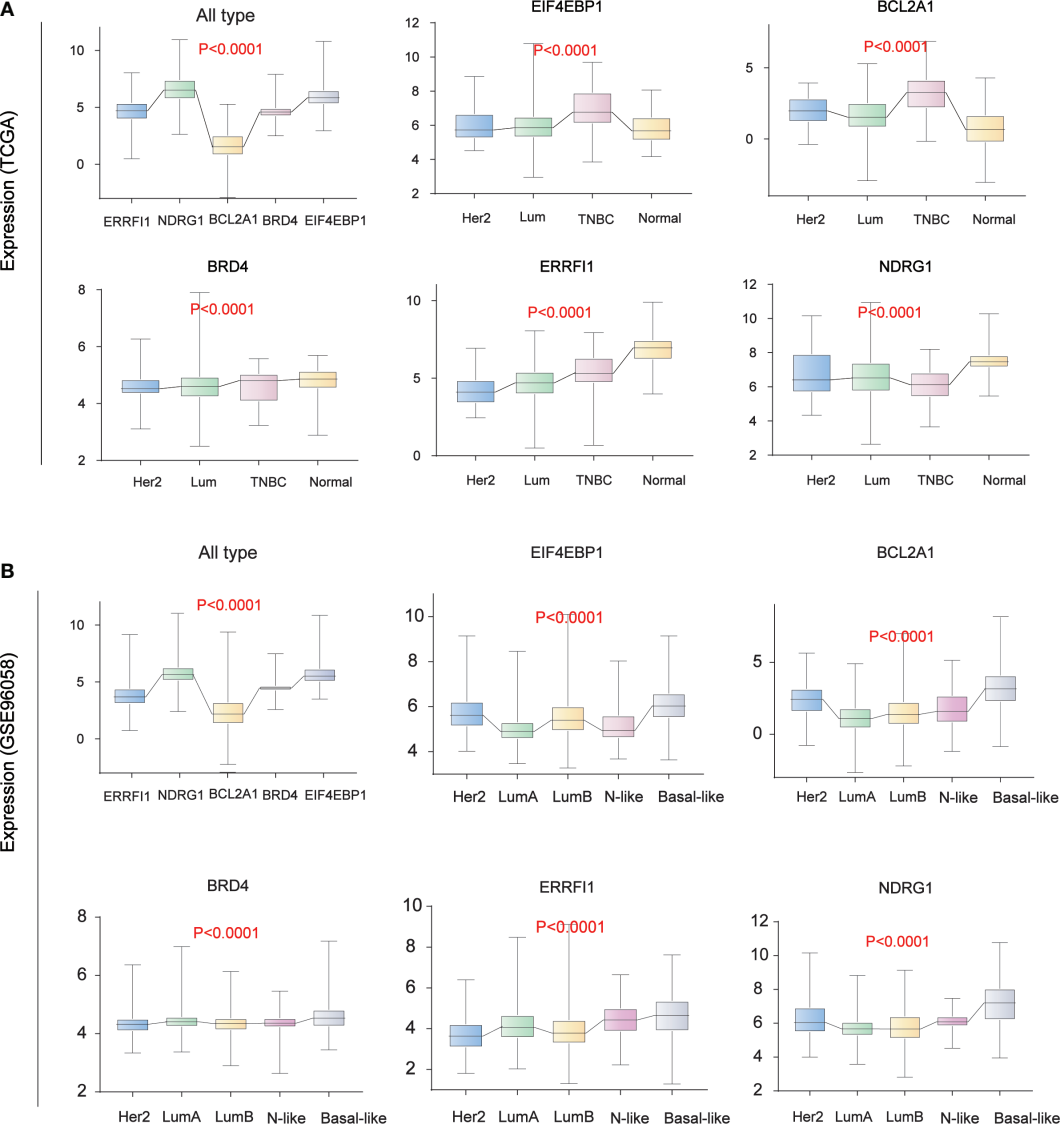
Breast Cancer Typing Expression. **(A)** Breast cancer typing expression in TCGA database. **(B)** Breast cancer typing expression in the GEO database (GSE96058).

We analyzed the expression levels of five core prognostic factors in two sets of BC classification data from public databases TCGA (**Figure 6A**) and GSE96058 (**Figure 6B**). The expression levels of EIF4EBP1, BCL2A1, NDRG1, ERRFI1 and BRD4 in all BC patients are consistent. The expression levels of EIF4EBP1 and BCL2A1 in BC patients are significantly higher than those in normal breast tissues, and with the increase of tumor malignancy. Its expression level increased.

### Vemurafenib impacts the expression of BCL2A1 by affecting PI3K-AKT signaling pathway and inhibit BC cell growth

**Figure 7:**
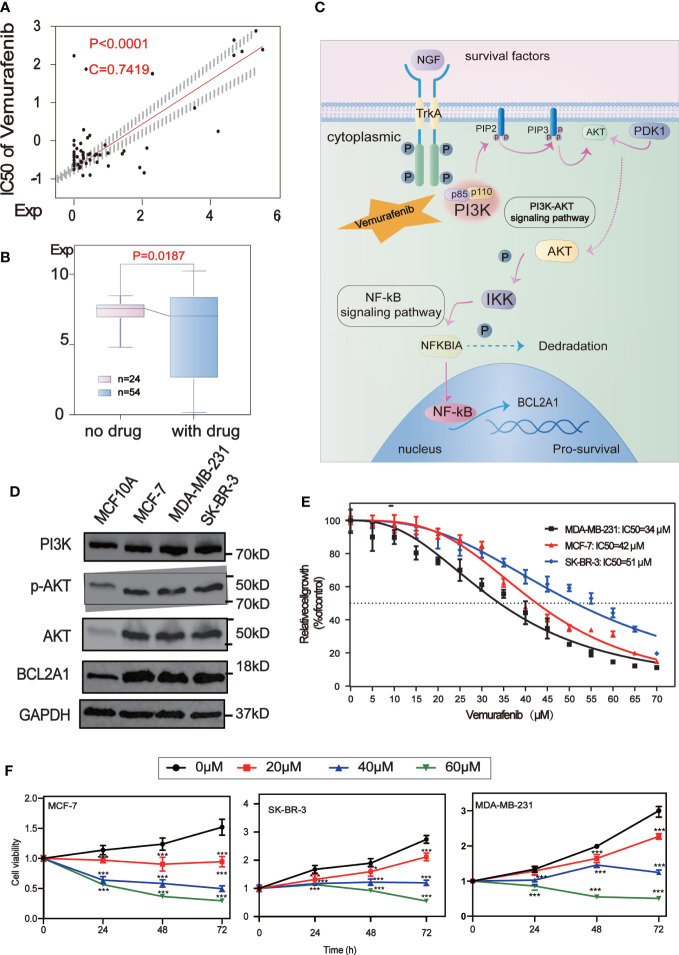
Drug sensitivity analysis of five central protein-coding genes. **(A)** Regulatory pathway map of BCL2A1 and the sensitive target of Vemurafenib. **(B)** The scatter plot shows the sensitivity analysis of BCL2A1 and the antitumor drug Vemurafenib. **(C)** Box-plot show the molecular formula of the Vemurafenib and the expression levels of BCL2A1 were analyzed using RNA sequencing data from 24 no drug and 54 with drug subjects in the GSE97681. **(D)** The expression levels of PI3K, AKT, P-Akt and BCL2A1 in MCF7, MDA-MB-231, SKBR3 and MCF10A cells were detected by Western Blot. **(E)** Vemurafenib inhibited cell viability in breast cancer cells. The concentration of vemurafenib resulting in 50% inhibition of control growth (IC50) was calculated. **(F)** Vemurafenib inhibit cell viability at different times. Vemurafenib (0, 20, 40, 60μM) inhibited MCF-7, SKBR3 and MDA-MB-231 cell viability at 0, 24, 48, and 72h. *P<0.05, **P<0.01, ***P<0.001.

Analysis of the potential correlation between the expression of 5 core protein coding genes and drug sensitivity in different human cancer cell lines from CellMiner database, which showed that the expression of BCL2A1 was positively correlated with the drug sensitivity of Vemurafenib (**Figure 7A**). The results of the correlation between other drugs and gene expression are placed in the **Supplementary Table** (**Table S2**). We used RNA sequencing data from 78 BC patients in GSE97681, 24 without and 54 with Vemurafenib, the expression levels of BCL2A1 were analyzed and were found to be significantly reduced with the drug (**Figure 7B**). A review of the literature and the KEGG database revealed a map of the regulatory pathways of Vemurafenib on BCL2A1-sensitive targets (19) (**Figure 7C**).

We first detected the expression levels of PI3K, AKT, p-Akt and BCL2A1 in MCF7, MDA-MB-231, SKBR3 and MCF10A cells by Western Blot analysis. It was confirmed that the expression of these proteins in BC cells (MCF7, MDA-MB-231, SKBR3) was higher than that in normal breast cells (MCF10A)(**Figure 7D**).

To explore the effect of vemurafenib on BC cells, the concentration of vemurafenib that resulted in 50% control growth inhibition (IC50) of MCF7, MDA-MB-231 and SKBR3 cells was assessed by MTT (**Figure 7E**). The IC50 of MCF-7 is 42 μM, MDA-MB-231 is 34 μM and SKBR3 is 51 μM. Next, the inhibitory activity of different concentrations of vemurafenib (0, 20, 40, 60 μM) on MCF7, SKBR3 and MDA-MB-231 cells at 0, 24, 48, and 72 h were further studied by MTT (**Figure 7F**). The results showed that vemurafenib inhibited cell proliferation in a dose-dependent manner, and the inhibitory effect increased with time.

**Figure 8:**
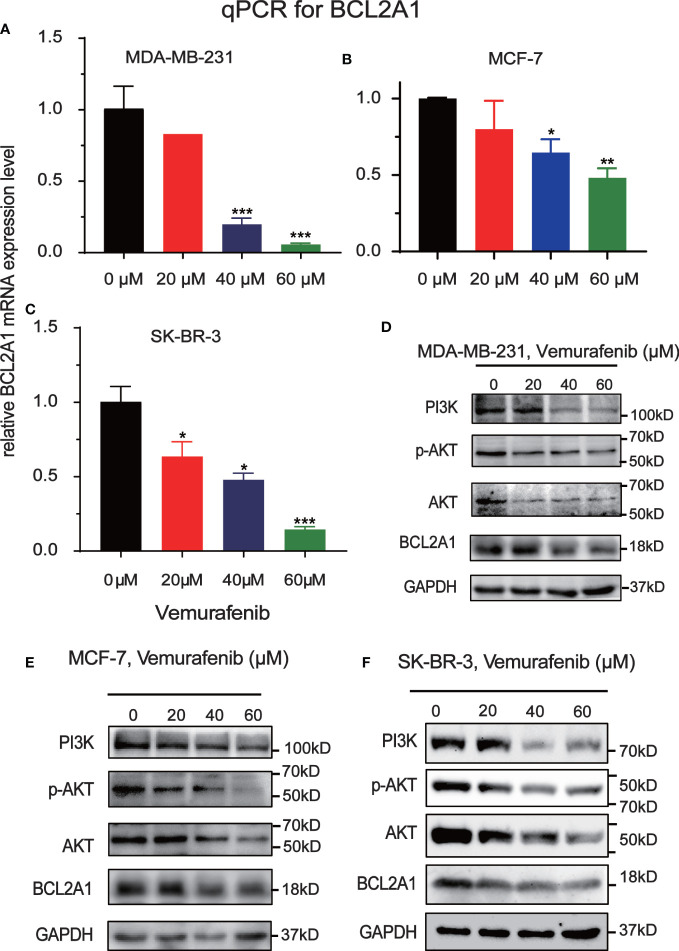
Inhibitory effect of Vemurafenib on different breast cancer cell lines. **(A)** Vemurafenib suppressed PI3K, AKT, p-AKT and BCL2A1 expression in MDA-MB-231cells. **(B)** Vemurafenib suppressed PI3K, AKT, p-AKT and BCL2A1 expression in MCF-7 cells. **(C)** Vemurafenib suppressed PI3K, AKT, p-AKT and BCL2A1 expression in SKBR3 cells. **(D)** Decreased levels of BCL2A1 in MDA-MB-231 cells treated with different concentrations of vemurafenib (0, 20, 40, 60 μM) were determined by RT-qPCR. **(E)** Decreased levels of BCL2A1 in MCF-7 cells treated with different concentrations of vemurafenib (0, 20, 40, 60 μM) were determined by RT-qPCR. **(F)** Decreased levels of BCL2A1 in SKBR3 cells treated with different concentrations of vemurafenib (0, 20, 40, 60 μM) were determined by RT-qPCR. (*P<0.05, **P<0.01, ***P<0.001.).

To further explore the mechanism by which vemurafenib inhibit the growth of MDA-MB-231, MCF7 and SKBR3 cells, the expression level of PI3K/AKT signaling pathway and BCL2A1 in vemurafenib treated cells was evaluated by western blot (**Figures 8A–C**). The expression level of PI3K, AKT, p-AKT and BCL2A1 were reduced in vemurafenib treated cells compared to without vemurafenib. This suggested that the drug inhibits the PI3K/AKT signaling pathway and BCL2A1. In addition, apoptosis-associated genes expression was detected. BCL2A1 downregulation was confirmed using RT-qPCR (**Figures 8D–F**), and the results indicated that vemurafenib promoted cell apoptosis of MDA-MB-231 and MCF-7.

## Discussion

BC is a malignant tumor that seriously endangers women’s health, and is occasionally seen in men. Most of the early symptoms of BC patients are not obvious and can be easily ignored, and the failure to seek timely medical attention leads to poor prognosis of BC. Personalized treatment and other new therapies can help patients choose the most appropriate treatment among various therapies. Establishing an accurate patient prognosis prediction system to provide patients with a more optimal treatment approach in molecularly targeted therapy is essential for personalized treatment (27). Molecular prognostic markers can change with tumor progression, and monitoring these markers can dynamically reflect patient prognosis. In addition, some molecular prognostic markers are involved in tumor progression and may be potential targets for tumor therapy and diagnostic indicators for early tumors (28). Molecular prognostic markers may be heterogeneous across patients, so that a group of molecular markers is superior to individual markers in terms of prognosis.

A large number of cancer and normal breast tissues were analyzed and screened in TCGA, GTEx, and GEO databases for broad applicability. 182 mouse immune escape related genes were obtained from Keith A. Lawson et al. and intersected with 2483 human immune escape related genes downloaded from the IMMPORT database, and 31 reliable immune escape related genes were identified. Fifteen of the immune escape related genes were significantly associated with survival and expression, and 181 differentially expressed proteins of the 15 hub genes were identified through the cBioportal database. Gene enrichment analysis showed that differentially expressed proteins are involved in many important biological processes, such as protein serine/threonine kinase activity, ubiquitin-like protein ligase binding, membrane raft, cell substrate connection, gland development and reproductive structure development. In addition, they are associated with important tumor-related pathways such as the PI3K-AKT signaling pathway, Hepatitis B, and Human papillomavirus infection pathway. The PI3K-AKT signaling pathway mainly inhibits cell apoptosis and promotes cell proliferation, indicating that immune escape genes play an important role in the occurrence and development of tumors. In recent years, an increasing number of studies had found that PI3K/Akt signaling pathway is closely related to the occurrence and development of lung cancer, BC, colorectal cancer, prostate cancer, ovarian cancer, liver cancer and lymphoma (29).

To find the most representative survival-related differentially expressed proteins, univariate Cox analysis, lasso regression analysis and multivariate Cox analysis were used to analyze the genes encoding these differentially expressed proteins. The prognostic model consisted of EIF4EBP1, BCL2A1, NDRG1, MRE11A, ERRFI1, CLDN7, PIK3CA, CASP9, BRD4, PDCD4 and G6PD. According to these 11 genetic characteristics, BC patients can be divided into high- and low-risk group, the survival rate of high-risk group is significantly lower than that of low-risk group. Differential expression analysis and survival analysis were performed on the prognostic models, and five core prognostic models were summarized, including EIF4EBP1, BCL2A1, NDRG1, ERRFI1 and BRD4. EIF4EBP1, NDRG1 and BRD4 are associated with poor prognosis of BC, while BCL2A1 and ERRFI1 are associated with good prognosis of BC. The nomogram found that the five prognostic factors had good prediction accuracy at 1, 3 and 5 years of BC. Similarly, HPA database (Human Protein Atlas) was used to test the performance of the prognostic model based on the characteristics of these five protein-coding genes, and the results showed that they performed well in predicting the prognosis of BC.

EIF4EBP1 (also known as 4EBP1) gene encodes a translation repressor protein, which competitively binds to eukaryotic translation initiation factor 4E (EIF4E), and inhibits the assembly of the EIF4E complex, thereby suppressing cap-dependent translation (30). In BC and cervical cancer, EIF4EBP1 is considered to be a major factor in the signaling pathway related to prognosis and malignancy, which was not changed by the presence of other upstream carcinogens (31, 32), it is consistent with our findings. The Bromodomain and extra-Terminal Domain (BET) protein BRD4 was a transcriptional and epigenetic regulator that plays a key role in embryonic and cancer development. Aberrant degradation of the BRD4 protein in cancer leads to resistance to BET inhibitors, so BRD4 is emerging as a promising anticancer therapeutic target (33). Recent evidence suggests that BRD4 has additional non-transcriptional functions in cancer, affecting processes such as DNA damage repair, checkpoint activation, or telomere homeostasis (34). ERBB receptor feedback inhibitor 1(ERRFI1) was known as a tumor suppressor, and initiates cell growth by directly inhibiting the epidermal growth factor receptor and its downstream pathway. In addition, ERFFI1 inhibits another receptor family (Erbb), whose activation leads to cell survival, proliferation, migration, and invasion (35). A dual mechanism by which ERRFI1 regulates AKT has been identified. ERRF1 inhibits growth and enhances response to chemotherapy in cells expressing high levels of EGFR. This was partly mediated by ERRFI1-dependent direct inhibition of the negative regulation of AKT signaling by EGFR. In cells expressing low levels of EGFR, ERRFI1 positively regulates AKT by interfering with the interaction of the inactivated phosphatase PHLPP with AKT, thereby promoting cell growth and chemotherapy desensitization (36). N-myc Downstream regulated gene-1 (NDRG1) was a potent inhibitor of metastasis regulated by hypoxia, metal ions including iron, free radicals nitric oxide (NO), and various stress stimuli. This intriguing molecule had shown multiple functions in cancer, regulating a plethora of oncogenes through cellular signaling and inhibiting epithelial mesenchymal transition (EMT), cell migration and angiogenesis (37).

BCL2A1 is a member of the Bcl-2-associated protein family, as an anti-apoptotic protein associated with resistance to chemotherapeutic drugs and targeted drugs (38). The Bcl-2 protein family plays a key role in regulating the internal pathways apoptosis by inhibiting cytochrome C and releasing activated proteases (39). We found that BCL2A1 expression was higher in BC than in normal breast tissue, and the expression of BCL2A1 was upregulated as the malignancy of the molecular subtype of BC increased. We also found patients with high expression BC showed a better survival advantage, which may result from the high correlation of BCL2A1 with immune cells and immune checkpoints (CYLA4, PD-1, PD-L1). However, the tissue clump-based transcriptome sequencing data are indistinguishable between cancer cells and their surrounding machinery cells, suggesting that if BCL2A1 expression is increased in the machinery cells surrounding tumor cells, this could also lead to an increase in overall BCL2A1 expression levels. As there is no effective drugs to treat cancers that high expression BCL2A1 at present (40), the discovery of its target drug could provided value to guide clinical treatment. We found a significant correlation between BCL2A1 and vemurafenib by drug sensitivity analysis. We also analyzed the data of GSE97681 and found that the expression level of BCL2A1was significantly lower in the group using vemurafenib compared to the non-drug group.

BCL2A1 is present downstream of PI3K/AKT and is regulated by it, affecting survival. Vemurafenib (PLX4032) is a novel small molecule BRAF inhibitor that has been approved by the US Food and Drug Administration for the treatment of patients with melanoma (41). One study found that applying Vemurafenib to a xenograft model of BC cells (MDA-MB-231) revealed that Vemurafenib treatment resulted in downregulation of PI3K-AKT signaling (19), which may be responsible for the downregulation of BCL2A1 expression after Vemurafenib. We found that vemurafenib had an inhibitory effect on the cell growth of MDA-MB-231, SKBR3 and MCF-7 cells by MTT assay. Further, we detected the level of PI3K/AKT signaling pathway in vemurafenib-treated cells by Western Blot and found that Vemurafenib significantly inhibited PI3K/AKT signaling pathway and the expression of BCL2A1. RT-qPCR confirmed the vemurafenib down-regulation of the expression of BCL2A1 too, and the results showed that vemurafenib inhibited the survival of MDA-MB-231, SKBR3 and MCF-7 cells.

In general, our study established an 11-gene prognostic model for predicting the efficacy of immunotherapy for BC, and further identified the 5 most core prognostic factors of the prognostic model, which plays a certain role in personalized treatment and accurate prognostic prediction for BC patients. In addition, this study also provides targeting reference drugs for the core prognostic genes to provide a reference for clinical practice, and the accuracy of drug targeting genes was verified by basic experiments and elucidates in detail that Vemurafenib suppresses BC by targeting the PI3K/AKT signaling pathway to inhibit the immune escape biomarker BCL2A1. However, further experimental studies and larger scale clinical trials are needed to further determine the applicability and accuracy of the 5 core factors that constitute prognostic markers for BC development and progression and the potential mechanisms of related drugs.

## Conclusion

In summary, our 11-gene expression prediction model based on multiple data sets is more economical and clinically feasible than whole-gene sequencing. We also plotted histograms of five core prognostic factor prediction models, which can individually assess the prognosis of different patients by detecting the expression of genes. We also elucidate in detail that Vemurafenib suppresses BC by targeting the PI3K/AKT signaling pathway to inhibit the immune escape biomarker BCL2A1. Our findings will provide therapeutic targets for individualized treatment of BC and find its potential drugs, which will certainly be more beneficial for selecting effective treatments.

## Data availability statement

The original contributions presented in the study are included in the article/**Supplementary Material**. Further inquiries can be directed to the corresponding author/s.

## Author contributions

ZX, QW, and JS contributed to the concept, design, and review of the study. YD and DZ organize the database, LYa and DZ carry out statistical analysis. LYa, LZ conducted experiments, DL, YD and AS wrote the first manuscripts. All authors were involved in revising, reading, and approving the version of the submitted manuscript.

## Funding

This work was supported by National Natural Science Foundation of China (No. 81972643, No. 82172962), Sichuan Science and Technology Project (2021YJ0201), Luzhou Science and Technology Bureau (2017LZXNYD-Z04), Sichuan Provincial Human Resources and Social Security Department (CW202002), Science and Technology Bureau of China (2017LZXNYD-Z04), Scientific Research Foundation for Doctors of the Affiliated Hospital of Southwest Medical University (20016) and Science and Technology Project founded by Southwest Medical University (2018- ZRZD-016).

## Conflict of interest

The authors declare that the research was conducted in the absence of any commercial or financial relationships that could be construed as a potential conflict of interest.

## Publisher’s note

All claims expressed in this article are solely those of the authors and do not necessarily represent those of their affiliated organizations, or those of the publisher, the editors and the reviewers. Any product that may be evaluated in this article, or claim that may be made by its manufacturer, is not guaranteed or endorsed by the publisher.

## Supplementary material

Figure S1 The remaining Kaplan – Meier survival curve in **Figure 1D** and **Figure 5A**.

Figure S2
**(A–E)** GSE42568 verify the risk model. **(F)** Cluster analysis of high and low risk groups and 28 types of immune cells.

## References

[1] Sung H Ferlay J Siegel RL Laversanne M Soerjomataram I Jemal A. Global cancer statistics 2020: GLOBOCAN estimates of incidence and mortality worldwide for 36 cancers in 185 countries. CA Cancer J Clin (2021) 71(3):209–49. doi: 10.3322/caac.21660 33538338

[2] Harbeck N Gnant M. Breast cancer. Lancet (2017) 389(10074):1134–50. doi: 10.1016/s0140-6736(16)31891-8 27865536

[3] Chen DS Mellman I. Elements of cancer immunity and the cancer-immune set point. Nature (2017) 541(7637):321–30. doi: 10.1038/nature21349 28102259

[4] Schreiber RD Old LJ Smyth MJ. Cancer immunoediting: integrating immunity's roles in cancer suppression and promotion. Science (2011) 331(6024):1565–70. doi: 10.1126/science.1203486 21436444

[5] De Jaeghere EA Denys HG De Wever O. Fibroblasts fuel immune escape in the tumor microenvironment. Trends Cancer (2019) 5(11):704–23. doi: 10.1016/j.trecan.2019.09.009 31735289

[6] Wang S Zhang Q Yu C Cao Y Zuo Y Yang L. Immune cell infiltration-based signature for prognosis and immunogenomic analysis in breast cancer. Brief Bioinform (2021) 22(2):2020–31. doi: 10.1093/bib/bbaa026 32141494

[7] Waks AG Winer EP. Breast cancer treatment: A review. Jama (2019) 321(3):288–300. doi: 10.1001/jama.2018.19323 30667505

[8] Emens LA. Breast cancer immunotherapy: Facts and hopes. Clin Cancer Res (2018) 24(3):511–20. doi: 10.1158/1078-0432.Ccr-16-3001 PMC5796849 28801472

[9] Jia H Truica CI Wang B Wang Y Ren X Harvey HA. Immunotherapy for triple-negative breast cancer: Existing challenges and exciting prospects. Drug Resist Update (2017) 32:1–15. doi: 10.1016/j.drup.2017.07.002 29145974

[10] Uno T Takeda K Kojima Y Yoshizawa H Akiba H Mittler RS. Eradication of established tumors in mice by a combination antibody-based therapy. Nat Med (2006) 12(6):693–8. doi: 10.1038/nm1405 16680149

[11] Smyth MJ Ngiow SF Ribas A Teng MW. Combination cancer immunotherapies tailored to the tumour microenvironment. Nat Rev Clin Oncol (2016) 13(3):143–58. doi: 10.1038/nrclinonc.2015.209 26598942

[12] Esteva FJ Hubbard-Lucey VM Tang J Pusztai L. Immunotherapy and targeted therapy combinations in metastatic breast cancer. Lancet Oncol (2019) 20(3):e175–e86. doi: 10.1016/s1470-2045(19)30026-9 30842061

[13] Network CGA:. Comprehensive molecular portraits of human breast tumours. Nature (2012) 490(7418):61–70. doi: 10.1038/nature11412 23000897 PMC3465532

[14] Ruggles KV Krug K Wang X Clauser KR Wang J Payne SH. Methods, tools and current perspectives in proteogenomics. Mol Cell Proteomics (2017) 16(6):959–81. doi: 10.1074/mcp.MR117.000024 PMC5461547 28456751

[15] Lawson KA Sousa CM Zhang X Kim E Akthar R Caumanns JJ. Functional genomic landscape of cancer-intrinsic evasion of killing by T cells. Nature (2020) 586(7827):120–6. doi: 10.1038/s41586-020-2746-2 PMC9014559 32968282

[16] O'Leary B Finn RS Turner NC. Treating cancer with selective CDK4/6 inhibitors. Nat Rev Clin Oncol (2016) 13(7):417–30. doi: 10.1038/nrclinonc.2016.26 27030077

[17] Wang R Yang Y Ye WW Xiang J Chen S Zou WB. Case report: Significant response to immune checkpoint inhibitor camrelizumab in a heavily pretreated advanced ER+/HER2- breast cancer patient with high tumor mutational burden. Front Oncol (2020) 10:588080. doi: 10.3389/fonc.2020.588080 33634015 PMC7900143

[18] Maughan KL Lutterbie MA Ham PS. Treatment of breast cancer. Am Fam Physician (2010) 81(11):1339–46. 20521754

[19] Zhang ZX Jin WJ Yang S Ji CL. BRAF kinase inhibitor exerts anti-tumor activity against breast cancer cells *via* inhibition of FGFR2. Am J Cancer Res (2016) 6(5):1040–52. PMC4889718 27293997

[20] Pircher M Winder T Trojan A. Response to vemurafenib in metastatic triple-negative breast cancer harbouring a BRAF V600E mutation: A case report and electronically captured patient-reported outcome. Case Rep Oncol (2021) 14(1):616–21. doi: 10.1159/000513905 PMC8077504 33976643

[21] Bhattacharya S Andorf S Gomes L Dunn P Schaefer H Pontius J. ImmPort: disseminating data to the public for the future of immunology. Immunol Res (2014) 58(2–3):234–9. doi: 10.1007/s12026-014-8516-1 24791905

[22] Gao J Aksoy BA Dogrusoz U Dresdner G Gross B Sumer SO. Integrative analysis of complex cancer genomics and clinical profiles using the cBioPortal. Sci Signal (2013) 6(269):pl1. doi: 10.1126/scisignal.2004088 23550210 PMC4160307

[23] Charoentong P Finotello F Angelova M Mayer C Efremova M Rieder D. Pan-cancer immunogenomic analyses reveal genotype-immunophenotype relationships and predictors of response to checkpoint blockade. Cell Rep (2017) 18(1):248–62. doi: 10.1016/j.celrep.2016.12.019 28052254

[24] Liu CJ Hu FF Xia MX Han L Zhang Q Guo AY. GSCALite: a web server for gene set cancer analysis. Bioinformatics (2018) 34(21):3771–2. doi: 10.1093/bioinformatics/bty411 29790900

[25] Li T Fan J Wang B Traugh N Chen Q Liu JS. TIMER: A web server for comprehensive analysis of tumor-infiltrating immune cells. Cancer Res (2017) 77(21):e108–e10. doi: 10.1158/0008-5472.Can-17-0307 PMC6042652 29092952

[26] Reinhold WC Sunshine M Liu H Varma S Kohn KW Morris J. CellMiner: a web-based suite of genomic and pharmacologic tools to explore transcript and drug patterns in the NCI-60 cell line set. Cancer Res (2012) 72(14):3499–511. doi: 10.1158/0008-5472.Can-12-1370 PMC3399763 22802077

[27] Jácome AA Coutinho AK Lima EM Andrade AC Dos Santos JS. Personalized medicine in gastric cancer: Where are we and where are we going? World J Gastroenterol (2016) 22(3):1160–71. doi: 10.3748/wjg.v22.i3.1160 PMC4716027 26811654

[28] Loumaye A Thissen JP. Biomarkers of cancer cachexia. Clin Biochem (2017) 50(18):1281–8. doi: 10.1016/j.clinbiochem.2017.07.011 28739222

[29] Lien EC Dibble CC Toker A. PI3K signaling in cancer: beyond AKT. Curr Opin Cell Biol (2017) 45:62–71. doi: 10.1016/j.ceb.2017.02.007 28343126 PMC5482768

[30] Gingras AC Raught B Sonenberg N. eIF4 initiation factors: effectors of mRNA recruitment to ribosomes and regulators of translation. Annu Rev Biochem (1999) 68:913–63. doi: 10.1146/annurev.biochem.68.1.913 10872469

[31] Rojo F Najera L Lirola J Jiménez J Guzmán M Sabadell MD. 4E-binding protein 1, a cell signaling hallmark in breast cancer that correlates with pathologic grade and prognosis. Clin Cancer Res (2007) 13(1):81–9. doi: 10.1158/1078-0432.Ccr-06-1560 17200342

[32] Castellvi J Garcia A Rojo F Ruiz-Marcellan C Gil A Baselga J. Phosphorylated 4E binding protein 1: a hallmark of cell signaling that correlates with survival in ovarian cancer. Cancer (2006) 107(8):1801–11. doi: 10.1002/cncr.22195 16983702

[33] Jin X Yan Y Wang D Ding D Ma T Ye Z. DUB3 promotes BET inhibitor resistance and cancer progression by deubiquitinating BRD4. Mol Cell (2018) 71(4):592–605.e4. doi: 10.1016/j.molcel.2018.06.036 30057199 PMC6086352

[34] Donati B Lorenzini E Ciarrocchi A. BRD4 and cancer: going beyond transcriptional regulation. Mol Cancer (2018) 17(1):164. doi: 10.1186/s12943-018-0915-9 30466442 PMC6251205

[35] Baselga J Swain SM. Novel anticancer targets: revisiting ERBB2 and discovering ERBB3. Nat Rev Cancer (2009) 9(7):463–75. doi: 10.1038/nrc2656 19536107

[36] Cairns J Fridley BL Jenkins GD Zhuang Y Yu J Wang L. Differential roles of ERRFI1 in EGFR and AKT pathway regulation affect cancer proliferation. EMBO Rep (2018) 19(3):e44767. doi: 10.15252/embr.201744767 29335246 PMC5835844

[37] Park KC Paluncic J Kovacevic Z Richardson DR. Pharmacological targeting and the diverse functions of the metastasis suppressor, NDRG1, in cancer. Free Radic Biol Med (2020) 157:154–75. doi: 10.1016/j.freeradbiomed.2019.05.020 31132412

[38] Vogler M. BCL2A1: the underdog in the BCL2 family. Cell Death Differ (2012) 19(1):67–74. doi: 10.1038/cdd.2011.158 22075983 PMC3252829

[39] García-Sáez AJ. The BCL-2 family saga. Nat Rev Mol Cell Biol (2020) 21(10):564–5. doi: 10.1038/s41580-020-0276-2 32699359

[40] Hiraki M Maeda T Mehrotra N Jin C Alam M Bouillez A. Targeting MUC1-c suppresses BCL2A1 in triple-negative breast cancer. Signal Transduct Target Ther (2018) 3:13. doi: 10.1038/s41392-018-0013-x 29760958 PMC5948210

[41] Hyman DM Puzanov I Subbiah V Faris JE Chau I Blay JY. Vemurafenib in multiple nonmelanoma cancers with BRAF V600 mutations. N Engl J Med (2015) 373(8):726–36. doi: 10.1056/NEJMoa1502309 PMC4971773 26287849

