# Supplementary figures and images for: Vemurafenib inhibits immune escape biomarker BCL2A1 by targeting PI3K/AKT signaling pathway to suppress breast cancer
Source: Front Oncol. 2022 Nov 29;12:906197. doi: 10.3389/fonc.2022.906197 (PMC9745811; doi:10.3389/fonc.2022.906197)

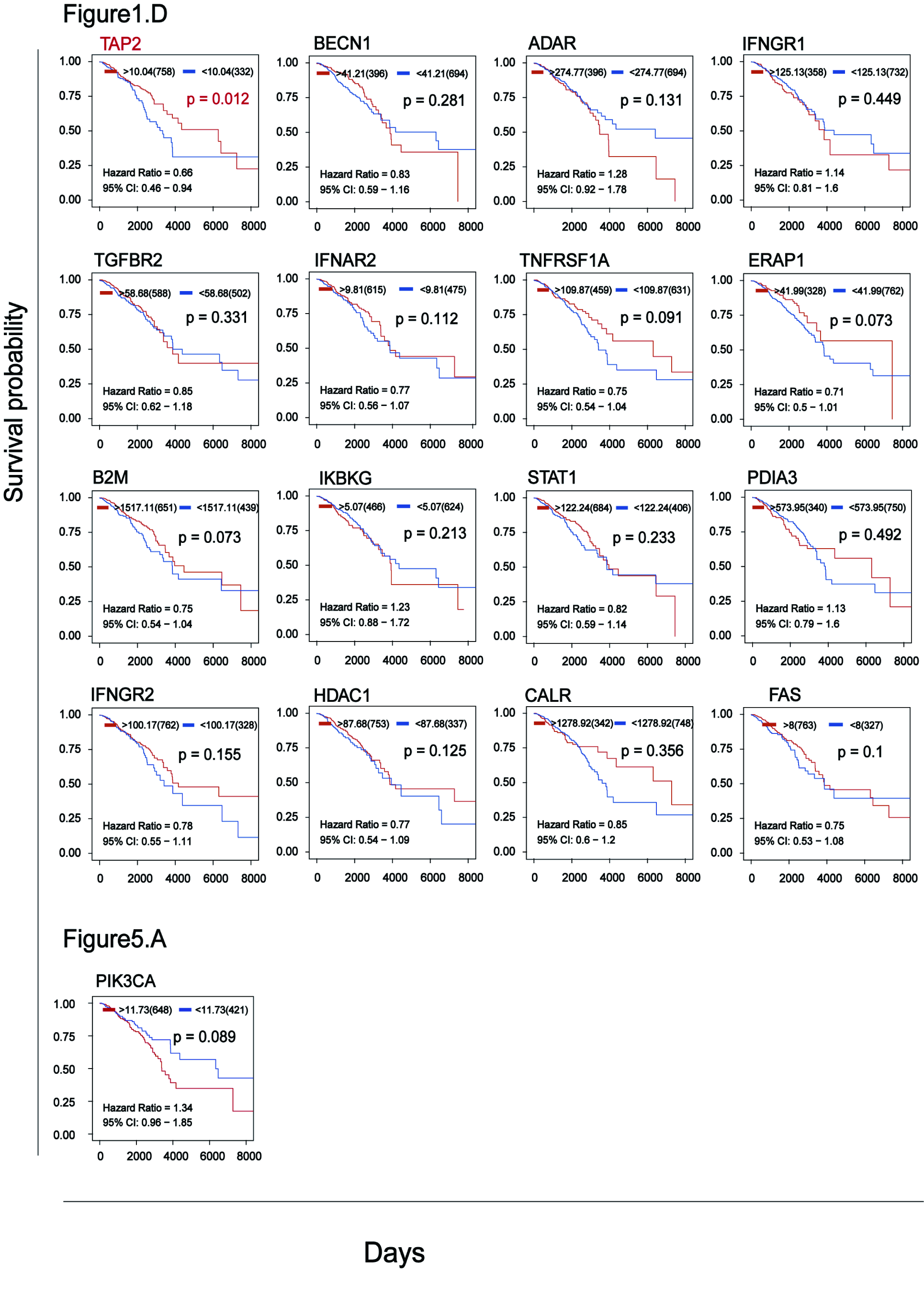

Supplement: Figure S1 — The remaining Kaplan – Meier survival curve in Figure 1D and Figure 5A . [file Image_1.tif]

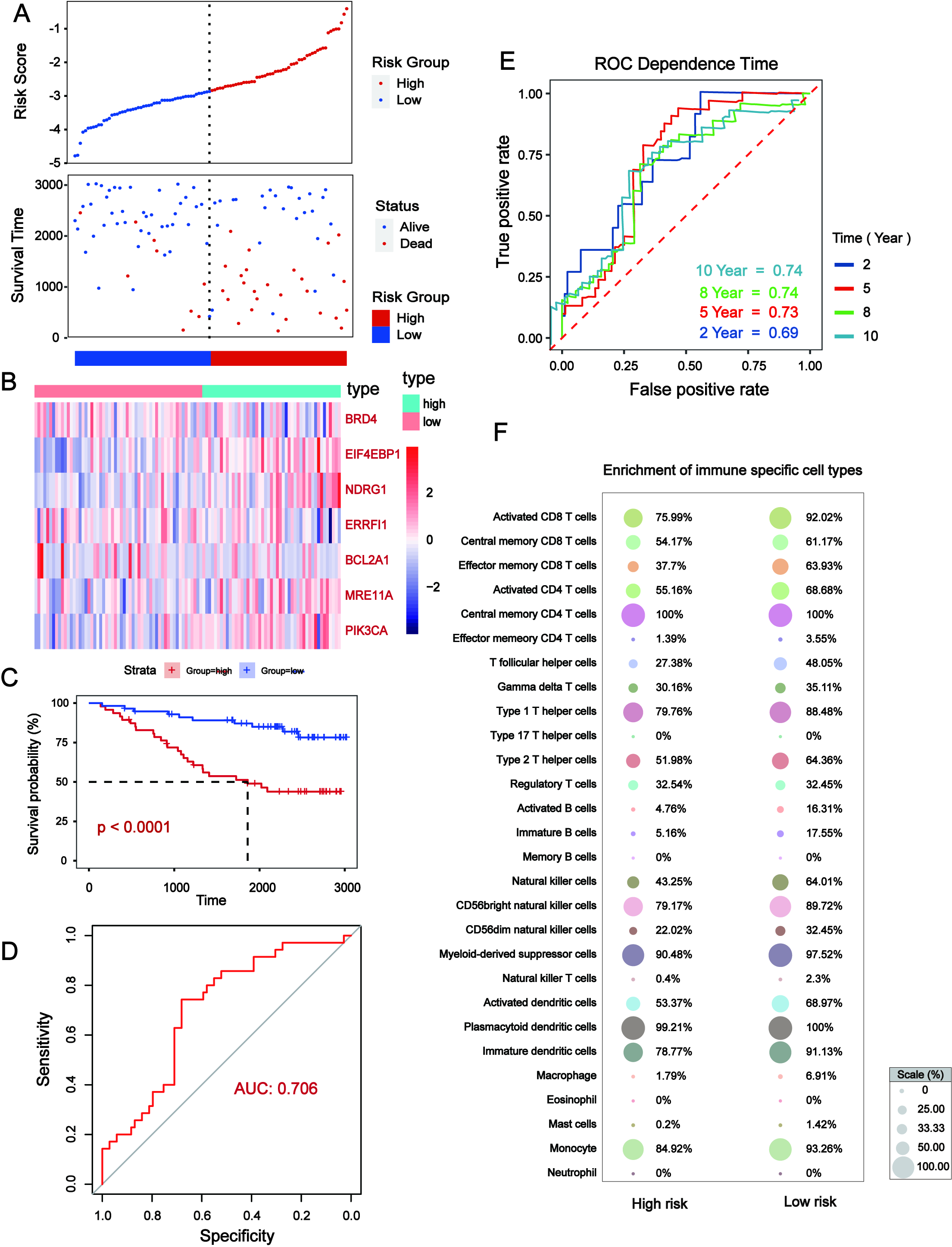

Supplement: Figure S2 — (A–E) GSE42568 verify the risk model. (F) Cluster analysis of high and low risk groups and 28 types of immune cells. [file Image_2.tif]
