# Supplementary material for: Vemurafenib inhibits immune escape biomarker BCL2A1 by targeting PI3K/AKT signaling pathway to suppress breast cancer
Source: Front Oncol. 2022 Nov 29;12:906197. doi: 10.3389/fonc.2022.906197 (PMC9745811; doi:10.3389/fonc.2022.906197)
Supplement: Supplementary file 3 [file Table_1.docx]

Table S1. 182 immune escape encoding genes

|  | name |  | name |  | name |  | mame |
| --- | --- | --- | --- | --- | --- | --- | --- |
| 1 | ATG10 | 51 | ANAPC15 | 101 | MED24 | 151 | ZC3H3 |
| 2 | ATG101 | 52 | ARF6 | 102 | MGAT1 | 152 | B2M |
| 3 | CFLAR | 53 | ATG14 | 103 | MTA2 | 153 | IFNGR1 |
| 4 | FITM2 | 54 | ATG7 | 104 | N6AMT1 | 154 | JAK2 |
| 5 | GPAA1 | 55 | ATG9A | 105 | NCBP1 | 155 | SOCS1 |
| 6 | GPI1 | 56 | ATP13A1 | 106 | NDUFAF6 | 156 | TAP1 |
| 7 | OTUD5 | 57 | ATXN7L3 | 107 | NPLOC4 | 157 | TAP2 |
| 8 | PTPN2 | 58 | BC003331 | 108 | NUP188 | 158 | TAPBP |
| 9 | RBCK1 | 59 | BCL2L1 | 109 | PCBP2 | 159 | ADAR |
| 10 | STAT2 | 60 | BOLA3 | 110 | PCED1B | 160 | IFNGR2 |
| 11 | TRAF2 | 61 | BRAT1 | 111 | PDCD6IP | 161 | IKBKG |
| 12 | ATG12 | 62 | BRPF1 | 112 | PDSS2 | 162 | IRF1 |
| 13 | ATG3 | 63 | CAD | 113 | PI4KB | 163 | JAK1 |
| 14 | ATG5 | 64 | CEP55 | 114 | PIGK | 164 | STAT1 |
| 15 | CHIC2 | 65 | CHMP5 | 115 | PIGT | 165 | TNFAIP3 |
| 16 | DNTTIP1 | 66 | COX6C | 116 | PKN2 | 166 | TNFRSF1A |
| 17 | EMC8 | 67 | CREBBP | 117 | PPP1CA | 167 | BECN1 |
| 18 | ERP44 | 68 | CUL3 | 118 | PPP1R8 | 168 | ERAP1 |
| 19 | FADD | 69 | DCP1A | 119 | PPP2R3C | 169 | HDAC1 |
| 20 | IST1 | 70 | DET1 | 120 | PRKCSH | 170 | PDIA3 |
| 21 | MAP3K7 | 71 | DICER1 | 121 | PSMB9 | 171 | PSMB8 |
| 22 | MEN1 | 72 | DNAJC13 | 122 | PSMG1 | 172 | TNFRSF1B |
| 23 | MOGS | 73 | DOT1L | 123 | PTAR1 | 173 | CALR |
| 24 | NDUFA13 | 74 | DPH5 | 124 | RCE1 | 174 | FAS |
| 25 | NXT1 | 75 | EIF3H | 125 | RGP1 | 175 | IFNAR1 |
| 26 | OTULIN | 76 | EMC2 | 126 | RIC1 | 176 | IFNAR2 |
| 27 | PIGS | 77 | EMC3 | 127 | S100PBP | 177 | IKBKB |
| 28 | PIGU | 78 | EMC4 | 128 | SARNP | 178 | IRF9 |
| 29 | PPP2R2A | 79 | EMC6 | 129 | SETD1A | 179 | MAPK1 |
| 30 | RB1CC1 | 80 | F8A | 130 | SETDB1 | 180 | TBK1 |
| 31 | RBM15 | 81 | FAM58B | 131 | SLC25A32 | 181 | TFRC |
| 32 | RIC8 | 82 | FNTB | 132 | SPCS1 | 182 | TGFBR2 |
| 33 | RNF31 | 83 | GALE | 133 | SRRT |  |  |
| 34 | SCAF4 | 84 | GLS | 134 | SRSF7 |  |  |
| 35 | SMG7 | 85 | H2-K1 | 135 | STOML2 |  |  |
| 36 | TAB1 | 86 | HCFC2 | 136 | SUSD6 |  |  |
| 37 | TAB2 | 87 | HDGFRP2 | 137 | TMEM208 |  |  |
| 38 | TMEM127 | 88 | HEXIM1 | 138 | TRADD |  |  |
| 39 | UBE2G2 | 89 | HIRA | 139 | TRPM7 |  |  |
| 40 | UBE2N | 90 | HSPA13 | 140 | UBR5 |  |  |
| 41 | VDAC2 | 91 | INO80 | 141 | UFC1 |  |  |
| 42 | VPS29 | 92 | IPPK | 142 | UFL1 |  |  |
| 43 | VPS35 | 93 | JAGN1 | 143 | USP7 |  |  |
| 44 | VPS4B | 94 | JMJD6 | 144 | UXS1 |  |  |
| 45 | WIPI2 | 95 | KAT6A | 145 | VPS13A |  |  |
| 46 | ZCCHC14 | 96 | KLF16 | 146 | VPS16 |  |  |
| 47 | ACAD9 | 97 | KMT2A | 147 | WDR7 |  |  |
| 48 | ACTB | 98 | LIPT2 | 148 | WDR83 |  |  |
| 49 | AGO2 | 99 | MED16 | 149 | WWP2 |  |  |
| 50 | AHSA1 | 100 | MED23 | 150 | YAP1 |  |  |
