# Supplementary material for: Vemurafenib inhibits immune escape biomarker BCL2A1 by targeting PI3K/AKT signaling pathway to suppress breast cancer
Source: Front Oncol. 2022 Nov 29;12:906197. doi: 10.3389/fonc.2022.906197 (PMC9745811; doi:10.3389/fonc.2022.906197)
Supplement: Supplementary file 4 [file Table_2.docx]

Table S2. The results of the correlation between drugs and gene expression.

| Gene | Drug | cor | pvalue |
| --- | --- | --- | --- |
| BCL2A1 | Vemurafenib | 0.741943997 | 1.78E-11 |
| BCL2A1 | PLX-4720 | 0.71501924 | 1.99E-10 |
| BCL2A1 | PLX-8394 | 0.712558648 | 2.45E-10 |
| BCL2A1 | Dabrafenib | 0.707515818 | 3.73E-10 |
| BCL2A1 | ARQ-680 | 0.703893175 | 5.01E-10 |
| BCL2A1 | TAK-632 | 0.675254092 | 4.46E-09 |
| BCL2A1 | BGB-283 | 0.648675567 | 2.76E-08 |
| BCL2A1 | SB-590885 | 0.644714321 | 3.57E-08 |
| BCL2A1 | MLN-2480 | 0.616900469 | 1.96E-07 |
| BCL2A1 | GDC-0994 | 0.561828151 | 3.66E-06 |
| BCL2A1 | CEP-32496 | 0.524948453 | 1.97E-05 |
| BCL2A1 | Encorafenib | 0.521846365 | 2.25E-05 |
| BCL2A1 | HYPOTHEMYCIN | 0.520523207 | 2.38E-05 |
| BCL2A1 | ulixertinib | 0.502866953 | 4.93E-05 |
| BCL2A1 | AZ-628 | 0.497942213 | 6.00E-05 |
| BCL2A1 | PD 184352 | 0.495915208 | 6.50E-05 |
| BCL2A1 | Refametinib | 0.482968583 | 0.000106929 |
| BCL2A1 | PD-98059 | 0.481376895 | 0.000113531 |
| BCL2A1 | LXH-254 | 0.475090572 | 0.000143429 |
| BCL2A1 | LY-3009120 | 0.457240405 | 0.000271917 |
| BCL2A1 | LY-3214996 | 0.451343615 | 0.000333361 |
| BCL2A1 | CC-90003 | 0.446805019 | 0.000388985 |
| BCL2A1 | Rebastinib | 0.435885418 | 0.000559 |
| BCL2A1 | Selumetinib | 0.430413832 | 0.000667367 |
| BCL2A1 | Bafetinib | 0.426748309 | 0.000750242 |
| BCL2A1 | SCH-772984 | 0.419310633 | 0.000947586 |
| BCL2A1 | AZD-0364 | 0.40977013 | 0.001268748 |
| BCL2A1 | ARRY-162 | 0.405826775 | 0.001427893 |
| BCL2A1 | RO-5126766 | 0.401874378 | 0.001605147 |
| BCL2A1 | Pimasertib | 0.39346609 | 0.002049269 |
| BCL2A1 | Cobimetinib (isomer 1) | 0.384015186 | 0.002676767 |
| BCL2A1 | MI-219 | 0.379677311 | 0.003018135 |
| BCL2A1 | ARRY-614 | 0.368948883 | 0.004033683 |
| BCL2A1 | SB-202190 | 0.359358233 | 0.0051858 |
| BCL2A1 | ABT-199 | 0.356917158 | 0.005521697 |
| BCL2A1 | Tipifarnib | 0.34692517 | 0.007103996 |
| BCL2A1 | (-)-Nutlin-3 | 0.343614111 | 0.007709324 |
| BCL2A1 | PD-0325901 | 0.334861881 | 0.009530814 |
| BCL2A1 | TAK-733 | 0.333002478 | 0.009962635 |
| BCL2A1 | RO-4987655 | 0.327926442 | 0.011228888 |
| BCL2A1 | MI-773 | 0.326440691 | 0.011624884 |
| BCL2A1 | AMG-232 | 0.326147129 | 0.011704538 |
| BCL2A1 | RAF-265 | 0.320229565 | 0.013413681 |
| BCL2A1 | Trametinib | 0.320086457 | 0.013457541 |
| BCL2A1 | Ixazomib citrate | 0.31123599 | 0.016420862 |
| BCL2A1 | PF-03758309 | 0.304309079 | 0.019113827 |
| BCL2A1 | milademetan | 0.301299836 | 0.020395874 |
| BCL2A1 | Okadaic acid | 0.297586277 | 0.02207795 |
| BCL2A1 | CGM-097 | 0.290572878 | 0.025576006 |
| BCL2A1 | HDM-201 | 0.289374204 | 0.026218252 |
| BCL2A1 | napabucasin | 0.28431158 | 0.029082418 |
| BCL2A1 | ARRY-704 | 0.282163114 | 0.03037478 |
| BCL2A1 | Hydrastinine HCl | 0.27898131 | 0.032376692 |
| BCL2A1 | UNC-0638 | 0.278701443 | 0.032557922 |
| BCL2A1 | RG-7388 | 0.277915076 | 0.033071673 |
| BCL2A1 | Rebimastat | 0.274251551 | 0.035554978 |
| BCL2A1 | PF-4942847 | 0.262753827 | 0.044373381 |
| BCL2A1 | Ixazomib | 0.262645785 | 0.044464066 |
| BCL2A1 | R-306465 | 0.258598997 | 0.047971943 |
| BCL2A1 | Abexinostat | 0.256519323 | 0.049860863 |
| BCL2A1 | MK-2461 | -0.257112659 | 0.049315863 |
| BCL2A1 | EGF-816 | -0.257988579 | 0.048520202 |
| BCL2A1 | BAY 61-3606 | -0.259526694 | 0.047148425 |
| BCL2A1 | Selonsertib | -0.26027125 | 0.046495886 |
| BCL2A1 | 6-Bromoindirubin-3'-Acetoxime | -0.263739725 | 0.04355286 |
| BCL2A1 | Midostaurin | -0.26450875 | 0.042921529 |
| BCL2A1 | IDH1-Comp 301 | -0.264919482 | 0.042587435 |
| BCL2A1 | Bisacodyl, active ingredient of Viraplex | -0.266830515 | 0.041061009 |
| BCL2A1 | CCT-251545 | -0.268062476 | 0.040101118 |
| BCL2A1 | CB-839 | -0.268947715 | 0.039422879 |
| BCL2A1 | Acetalax | -0.2705676 | 0.038206324 |
| BCL2A1 | PF-2771 | -0.275250778 | 0.034862775 |
| BCL2A1 | SW-044248 | -0.275271166 | 0.03484877 |
| BCL2A1 | Plinabulin | -0.282376378 | 0.030244386 |
| BCL2A1 | Fostamatinib | -0.284370374 | 0.029047709 |
| BCL2A1 | ON-123300 | -0.288866399 | 0.026494394 |
| BCL2A1 | Dasatinib | -0.293110246 | 0.024260098 |
| BCL2A1 | AS-703569 | -0.293346312 | 0.024140631 |
| BCL2A1 | VE-821 | -0.295282764 | 0.023179243 |
| BCL2A1 | OTS-964 | -0.295587516 | 0.023030931 |
| BCL2A1 | BAL-101553 | -0.303232676 | 0.019564305 |
| BCL2A1 | Sabutoclax | -0.306823357 | 0.018095857 |
| BCL2A1 | Fedratinib | -0.312132227 | 0.016097366 |
| BCL2A1 | BLU-667 | -0.312905662 | 0.015822594 |
| BCL2A1 | Saracatinib | -0.320608145 | 0.013298246 |
| BCL2A1 | CCT-128930 | -0.320817006 | 0.013234927 |
| BCL2A1 | PYRAZOLOACRIDINE | -0.322658839 | 0.012687694 |
| BCL2A1 | BAY-876 | -0.328558479 | 0.011064 |
| BCL2A1 | SAR-20347 | -0.330492745 | 0.010572321 |
| BCL2A1 | Lexibulin | -0.335534144 | 0.009378738 |
| BCL2A1 | Docetaxel | -0.336538089 | 0.009155554 |
| BCL2A1 | brigatinib | -0.342408081 | 0.007940731 |
| BCL2A1 | SCH-1473759 | -0.343388197 | 0.007752219 |
| BCL2A1 | dimethylfasudil | -0.34361037 | 0.007710033 |
| BCL2A1 | ABC-1183 | -0.346953959 | 0.00709892 |
| BCL2A1 | BMS-690514 | -0.357169177 | 0.005486156 |
| BCL2A1 | RX-5902 | -0.373384004 | 0.003582078 |
| BCL2A1 | umbralisib | -0.377484368 | 0.003204991 |
| BCL2A1 | LY-2874455 | -0.391816927 | 0.002148258 |
| BCL2A1 | ARV-825 | -0.40597605 | 0.001421557 |
| BCL2A1 | Ombrabulin | -0.413381528 | 0.001137179 |
| BCL2A1 | Varbulin | -0.437179909 | 0.000535817 |
| BCL2A1 | TPX-0005 | -0.443023381 | 0.000441638 |
| BRD4 | P-529 | 0.36573866 | 0.004391241 |
| BRD4 | Deforolimius | 0.338379351 | 0.008758201 |
| BRD4 | LGK-974 | 0.332505371 | 0.010080915 |
| BRD4 | OSI-027 | 0.325661096 | 0.011837451 |
| BRD4 | XL-147 | 0.324434095 | 0.012178804 |
| BRD4 | Everolimus | 0.312484606 | 0.015971677 |
| BRD4 | GSK-2126458 | 0.303022987 | 0.019653103 |
| BRD4 | SAR-245409 | 0.301366906 | 0.020366523 |
| BRD4 | 5-Fluoro deoxy uridine 10mer | 0.298809776 | 0.021511241 |
| BRD4 | AZD-5363 | 0.298267526 | 0.021760865 |
| BRD4 | Rapamycin | 0.290728014 | 0.025493861 |
| BRD4 | PI-103 | 0.287277303 | 0.02737439 |
| BRD4 | (+)-JQ1 | 0.286707722 | 0.027695725 |
| BRD4 | 6-(4-pyrimidinyl)-1H-indazole derivative | 0.277485522 | 0.03335515 |
| BRD4 | FH-535 | 0.276993061 | 0.033682628 |
| BRD4 | RAPAMYCIN | 0.273165875 | 0.036319964 |
| BRD4 | PF-04691502 | 0.268548622 | 0.039727467 |
| BRD4 | MK-2206 | 0.267853309 | 0.040262773 |
| BRD4 | PKM2 (9) | 0.266810863 | 0.041076473 |
| BRD4 | EMD-1204831 | 0.263931696 | 0.043394551 |
| BRD4 | cs-1730 | 0.260908053 | 0.04594367 |
| BRD4 | PF-06463922 | -0.257015955 | 0.049404356 |
| BRD4 | ON-123300 | -0.26164681 | 0.045309792 |
| BRD4 | Ribavirin | -0.263005216 | 0.044162964 |
| BRD4 | LDK-378 | -0.266625561 | 0.041222524 |
| BRD4 | TAE-684 | -0.270512311 | 0.038247329 |
| BRD4 | Oxaliplatin | -0.272138803 | 0.037056175 |
| BRD4 | AM-5992 | -0.272142639 | 0.037053403 |
| BRD4 | KW-2449 | -0.287115791 | 0.027465189 |
| BRD4 | Danusertib | -0.293140262 | 0.02424488 |
| BRD4 | AMINOFLAVONE | -0.294611248 | 0.023508898 |
| BRD4 | Palbociclib | -0.297694732 | 0.02202721 |
| BRD4 | EXEL-2280 | -0.298446553 | 0.02167818 |
| BRD4 | PF-06873600 | -0.301012562 | 0.020521993 |
| BRD4 | CC-671 | -0.301738901 | 0.020204386 |
| BRD4 | CX-5461 | -0.309808156 | 0.016947716 |
| BRD4 | CCT-271850 | -0.365216529 | 0.004451966 |
| BRD4 | CG-806 | -0.377745023 | 0.003182257 |
| BRD4 | BOS-172722 | -0.399112365 | 0.001740462 |
| EIF4EBP1 | SNS-314 | 0.447290527 | 0.000382655 |
| EIF4EBP1 | Quizartinib | 0.398965084 | 0.001747956 |
| EIF4EBP1 | Floxuridine | 0.375687615 | 0.003365662 |
| EIF4EBP1 | Fenretinide | 0.344174354 | 0.007603844 |
| EIF4EBP1 | Lapachone | 0.343134384 | 0.007800658 |
| EIF4EBP1 | Seliciclib | 0.332942189 | 0.009976915 |
| EIF4EBP1 | Zoledronate | 0.307507172 | 0.017827126 |
| EIF4EBP1 | (+)-JQ1 | 0.307096666 | 0.017988039 |
| EIF4EBP1 | Indibulin | 0.303992853 | 0.019245244 |
| EIF4EBP1 | FENRETINIDE | 0.295520513 | 0.02306347 |
| EIF4EBP1 | Azacitidine | 0.294796454 | 0.023417585 |
| EIF4EBP1 | Axitinib | 0.290442248 | 0.025645347 |
| EIF4EBP1 | ABBV-075 | 0.289201568 | 0.026311858 |
| EIF4EBP1 | 5-Fluoro deoxy uridine 10mer | 0.288728356 | 0.026569883 |
| EIF4EBP1 | Sabutoclax | 0.286877634 | 0.027599537 |
| EIF4EBP1 | LY-2801653 | 0.285244379 | 0.028535829 |
| EIF4EBP1 | EMD-1204831 | 0.281279759 | 0.030919899 |
| EIF4EBP1 | Curcumin | 0.280509453 | 0.031401904 |
| EIF4EBP1 | pyridoclax | 0.278658427 | 0.032585853 |
| EIF4EBP1 | AZD-3514 | 0.273323079 | 0.036208357 |
| EIF4EBP1 | Benzimate | 0.27177864 | 0.037317254 |
| EIF4EBP1 | UMI-77 | 0.270273698 | 0.038424712 |
| EIF4EBP1 | AZACITIDINE | 0.265202194 | 0.04235872 |
| EIF4EBP1 | Hydrastinine HCl | 0.264780478 | 0.042700262 |
| EIF4EBP1 | ZM-336372 | 0.260364258 | 0.046414895 |
| EIF4EBP1 | cs-1730 | 0.260193576 | 0.046563613 |
| EIF4EBP1 | S-49076 | 0.25919463 | 0.047441859 |
| EIF4EBP1 | Acetalax | 0.259008323 | 0.047607145 |
| EIF4EBP1 | Cladribine | 0.257868421 | 0.048628725 |
| EIF4EBP1 | Vandetanib | -0.264495193 | 0.042932593 |
| EIF4EBP1 | CEP-37440 | -0.27854836 | 0.03265741 |
| EIF4EBP1 | Neratinib | -0.299147743 | 0.021356889 |
| EIF4EBP1 | PF-06873600 | -0.305949337 | 0.018444357 |
| EIF4EBP1 | AZD-3463 | -0.306627581 | 0.018173427 |
| EIF4EBP1 | EXEL-7647 | -0.329417984 | 0.010843131 |
| EIF4EBP1 | brigatinib | -0.339234633 | 0.008578786 |
| ERRFI1 | BLU-667 | 0.37955585 | 0.003028225 |
| ERRFI1 | entosplenitib | 0.360124253 | 0.005084165 |
| ERRFI1 | Irofulven | 0.322692926 | 0.012677753 |
| ERRFI1 | Kahalide F | 0.310504807 | 0.016688884 |
| ERRFI1 | TPX-0005 | 0.298045479 | 0.02186379 |
| ERRFI1 | Staurosporine | 0.289911567 | 0.025928676 |
| ERRFI1 | Simvastatin | 0.271975171 | 0.037174602 |
| ERRFI1 | Oxaliplatin | -0.256412241 | 0.049959743 |
| ERRFI1 | 5-Fluoro deoxy uridine 10mer | -0.256482044 | 0.049895268 |
| ERRFI1 | AMG-900 | -0.257253426 | 0.049187281 |
| ERRFI1 | AZD-2858 | -0.257530667 | 0.048934837 |
| ERRFI1 | Dabrafenib | -0.257535092 | 0.048930816 |
| ERRFI1 | CUDC-305 | -0.258274652 | 0.048262626 |
| ERRFI1 | Tamoxifen | -0.258576409 | 0.047992141 |
| ERRFI1 | CNDAC | -0.258815152 | 0.047779019 |
| ERRFI1 | Imatinib | -0.258984964 | 0.047627902 |
| ERRFI1 | Cytarabine | -0.259040615 | 0.047578463 |
| ERRFI1 | Doxorubicin | -0.259371117 | 0.047285717 |
| ERRFI1 | 6-THIOGUANINE | -0.260785891 | 0.046049186 |
| ERRFI1 | BGB-283 | -0.261150808 | 0.045734581 |
| ERRFI1 | Panobinostat | -0.261443692 | 0.045483354 |
| ERRFI1 | Ixabepilone | -0.261472208 | 0.045458955 |
| ERRFI1 | AZD-3514 | -0.26237706 | 0.044690282 |
| ERRFI1 | Sulfatinib | -0.263197815 | 0.044002311 |
| ERRFI1 | Sapacitabine | -0.263550728 | 0.04370918 |
| ERRFI1 | cs-1730 | -0.263633475 | 0.043640683 |
| ERRFI1 | Voreloxin | -0.263650767 | 0.04362638 |
| ERRFI1 | Vemurafenib | -0.266198503 | 0.041560751 |
| ERRFI1 | AMG-232 | -0.267052494 | 0.040886667 |
| ERRFI1 | AZD-5153 | -0.269366316 | 0.039105478 |
| ERRFI1 | CPI-0610 | -0.26956031 | 0.038959102 |
| ERRFI1 | SNX-5422 | -0.270271219 | 0.038426558 |
| ERRFI1 | PWT-33597 | -0.270368475 | 0.038354174 |
| ERRFI1 | DAUNORUBICIN | -0.270572841 | 0.038202439 |
| ERRFI1 | ARQ-680 | -0.270590352 | 0.03818946 |
| ERRFI1 | CGM-097 | -0.27111582 | 0.037801707 |
| ERRFI1 | CEP-14083 | -0.272142838 | 0.037053259 |
| ERRFI1 | AZD-3965 | -0.272671362 | 0.036672905 |
| ERRFI1 | HPI-1 | -0.273479714 | 0.036097437 |
| ERRFI1 | milademetan | -0.273618308 | 0.035999529 |
| ERRFI1 | JZL-195 | -0.274196678 | 0.035593319 |
| ERRFI1 | delanzomib | -0.274745467 | 0.03521141 |
| ERRFI1 | TESTOLACTONE | -0.275701304 | 0.034554363 |
| ERRFI1 | Saridegib | -0.275827932 | 0.034468088 |
| ERRFI1 | CEP-9722 | -0.27591481 | 0.034408999 |
| ERRFI1 | LGK-974 | -0.277493952 | 0.033349567 |
| ERRFI1 | ABT-199 | -0.277598022 | 0.033280712 |
| ERRFI1 | Dromostanolone Propionate | -0.278529592 | 0.032669624 |
| ERRFI1 | RG-7112 | -0.278692349 | 0.032563826 |
| ERRFI1 | Parthenolide | -0.279655188 | 0.031943766 |
| ERRFI1 | ABT-737 | -0.279913327 | 0.031779212 |
| ERRFI1 | tfdu | -0.280175042 | 0.0316131 |
| ERRFI1 | BN-2629 | -0.280325421 | 0.031517982 |
| ERRFI1 | tic10 | -0.280552323 | 0.031374915 |
| ERRFI1 | Hydrastinine HCl | -0.280632713 | 0.031324357 |
| ERRFI1 | Tipifarnib | -0.283603 | 0.029503459 |
| ERRFI1 | Mocetinostat | -0.285423762 | 0.02843171 |
| ERRFI1 | Chelerythrine | -0.286385521 | 0.027878895 |
| ERRFI1 | FENRETINIDE | -0.286505639 | 0.02781049 |
| ERRFI1 | Dexrazoxane | -0.290140403 | 0.02580618 |
| ERRFI1 | Vorinostat | -0.291469014 | 0.025104557 |
| ERRFI1 | Thiotepa | -0.292702546 | 0.024467601 |
| ERRFI1 | Bafetinib | -0.293708972 | 0.023958064 |
| ERRFI1 | CCT-251545 | -0.294002327 | 0.023811239 |
| ERRFI1 | Axitinib | -0.294216705 | 0.023704424 |
| ERRFI1 | Lomustine | -0.295138379 | 0.023249792 |
| ERRFI1 | Nelarabine | -0.295950329 | 0.022855408 |
| ERRFI1 | Triethylenemelamine | -0.297512818 | 0.022112374 |
| ERRFI1 | Estramustine | -0.298127036 | 0.021825938 |
| ERRFI1 | Fluphenazine | -0.298162261 | 0.021809607 |
| ERRFI1 | MI-773 | -0.299566389 | 0.021166994 |
| ERRFI1 | Pevonedistat | -0.301806431 | 0.02017507 |
| ERRFI1 | Buparlisib | -0.303059483 | 0.019637623 |
| ERRFI1 | MK-8033 | -0.307879995 | 0.01768205 |
| ERRFI1 | Uracil mustard | -0.311115003 | 0.016464955 |
| ERRFI1 | RG-7388 | -0.311128175 | 0.01646015 |
| ERRFI1 | Amuvatinib | -0.312221018 | 0.016065616 |
| ERRFI1 | MI-219 | -0.312670915 | 0.015905563 |
| ERRFI1 | HYPOTHEMYCIN | -0.313027575 | 0.015779651 |
| ERRFI1 | Valrubicin | -0.317444701 | 0.014289629 |
| ERRFI1 | Ixazomib | -0.319576476 | 0.013614845 |
| ERRFI1 | Chlorambucil | -0.320193269 | 0.013424794 |
| ERRFI1 | I-BET-151 | -0.320808365 | 0.013237541 |
| ERRFI1 | GDC-0339 | -0.321325282 | 0.01308192 |
| ERRFI1 | Epirubicin | -0.322763167 | 0.012657289 |
| ERRFI1 | AFP464 | -0.324800174 | 0.012076083 |
| ERRFI1 | Carboplatin | -0.327144742 | 0.011435756 |
| ERRFI1 | HDM-201 | -0.328545266 | 0.011067426 |
| ERRFI1 | Teniposide | -0.329028052 | 0.010942855 |
| ERRFI1 | Isotretinoin | -0.330922346 | 0.010465723 |
| ERRFI1 | Nilotinib | -0.332829212 | 0.010003724 |
| ERRFI1 | DMAPT | -0.334937863 | 0.009513519 |
| ERRFI1 | Nitrogen mustard | -0.336459584 | 0.009172839 |
| ERRFI1 | R-306465 | -0.336611561 | 0.009139404 |
| ERRFI1 | Hydroxyurea | -0.339158929 | 0.008594537 |
| ERRFI1 | GSK-2194069 | -0.341750697 | 0.008069398 |
| ERRFI1 | Bendamustine | -0.342379796 | 0.00794623 |
| ERRFI1 | Denileukin Diftitox Ontak | -0.343651946 | 0.007702161 |
| ERRFI1 | Pipobroman | -0.349956197 | 0.006586701 |
| ERRFI1 | Carmustine | -0.353389241 | 0.006040881 |
| ERRFI1 | Idarubicin | -0.359572779 | 0.005157155 |
| ERRFI1 | S-64315 | -0.359787069 | 0.005128684 |
| ERRFI1 | AMG-176 | -0.367679115 | 0.004171914 |
| ERRFI1 | Raloxifene | -0.370075336 | 0.003914458 |
| ERRFI1 | ARRY-614 | -0.376225954 | 0.003316789 |
| ERRFI1 | Arsenic trioxide | -0.376564894 | 0.003286343 |
| ERRFI1 | JNJ-54302833 | -0.378415288 | 0.003124451 |
| ERRFI1 | Entinostat | -0.386468197 | 0.002499326 |
| ERRFI1 | Melphalan | -0.390367073 | 0.002238784 |
| ERRFI1 | Etoposide | -0.392791866 | 0.002089235 |
| ERRFI1 | AZD-5991 | -0.400070405 | 0.001692412 |
| ERRFI1 | DACARBAZINE | -0.411666788 | 0.001198037 |
| ERRFI1 | XK-469 | -0.42110992 | 0.000895974 |
| ERRFI1 | SB-202190 | -0.442663337 | 0.000446975 |
| ERRFI1 | S-63845 | -0.448667397 | 0.000365212 |
| ERRFI1 | pyridoclax | -0.458934188 | 0.000256287 |
| ERRFI1 | Imexon | -0.4646534 | 0.000209371 |
| ERRFI1 | Cyclophosphamide | -0.485355773 | 9.77E-05 |
| NDRG1 | Milciclib | 0.453938952 | 0.000304909 |
| NDRG1 | GSK-2636771 | 0.45361024 | 0.000308386 |
| NDRG1 | BMS-911543 | 0.442792088 | 0.00044506 |
| NDRG1 | Motesanib | 0.441941747 | 0.000457846 |
| NDRG1 | Silmitasertib | 0.430088584 | 0.00067437 |
| NDRG1 | AMG-319 | 0.417916788 | 0.000989394 |
| NDRG1 | MK-2461 | 0.413472878 | 0.001134016 |
| NDRG1 | Fostamatinib | 0.407159601 | 0.001372205 |
| NDRG1 | SAR-260301 (enantiomer 1) | 0.402667725 | 0.001568063 |
| NDRG1 | entosplenitib | 0.393278819 | 0.002060301 |
| NDRG1 | AZD-5363 | 0.392770169 | 0.002090533 |
| NDRG1 | JNJ-42756493 | 0.387801986 | 0.002407308 |
| NDRG1 | futibutinib | 0.381426505 | 0.00287609 |
| NDRG1 | AZD-1480 | 0.37990233 | 0.002999519 |
| NDRG1 | PRN-1371 | 0.378833307 | 0.003088874 |
| NDRG1 | P-529 | 0.370535907 | 0.003866615 |
| NDRG1 | spebrutinib | 0.370517939 | 0.003868471 |
| NDRG1 | AZD-1208 | 0.367220632 | 0.004222844 |
| NDRG1 | E-3810 | 0.367212477 | 0.004223755 |
| NDRG1 | AZD-7762 | 0.36201576 | 0.004840672 |
| NDRG1 | TP-3654 | 0.36089138 | 0.004984144 |
| NDRG1 | ASP-5878 | 0.359648825 | 0.005147036 |
| NDRG1 | Defactinib | 0.357786157 | 0.005399992 |
| NDRG1 | enantiomer of PF-4176340 | 0.35714233 | 0.005489932 |
| NDRG1 | ENMD-2076 Precursor | 0.356118999 | 0.005635596 |
| NDRG1 | BLU-667 | 0.356105956 | 0.005637474 |
| NDRG1 | Ipatasertib | 0.351230971 | 0.006379185 |
| NDRG1 | 6-Bromoindirubin-3'-Acetoxime | 0.350562993 | 0.006487197 |
| NDRG1 | KU-55933 | 0.338710587 | 0.008688333 |
| NDRG1 | Simvastatin | 0.335775558 | 0.009324643 |
| NDRG1 | Avagacestat | 0.332225859 | 0.010147955 |
| NDRG1 | INCB-047775 | 0.330106027 | 0.010669081 |
| NDRG1 | Idelalisib | 0.327886537 | 0.01123937 |
| NDRG1 | WORTMANNIN | 0.324670498 | 0.012112384 |
| NDRG1 | CCT-128930 | 0.323672445 | 0.012394939 |
| NDRG1 | Quercetin | 0.320173542 | 0.013430837 |
| NDRG1 | 4SC-202 | 0.319091088 | 0.01376603 |
| NDRG1 | IDH1-Comp 301 | 0.318460016 | 0.013964747 |
| NDRG1 | IDH-C227 | 0.318285127 | 0.014020251 |
| NDRG1 | AZD-4547 | 0.315680593 | 0.014869553 |
| NDRG1 | SAR-245409 | 0.315329188 | 0.014987452 |
| NDRG1 | Cabozantinib | 0.314265578 | 0.015349197 |
| NDRG1 | AZD-8186 | 0.311670636 | 0.01626329 |
| NDRG1 | M2698 | 0.304323547 | 0.019107833 |
| NDRG1 | LY-294002 | 0.304075478 | 0.019210833 |
| NDRG1 | GSK-2141795 | 0.30331059 | 0.019531398 |
| NDRG1 | Dovitinib | 0.302629987 | 0.019820451 |
| NDRG1 | GSK-2606414 | 0.299509697 | 0.021192625 |
| NDRG1 | ZSTK-474 | 0.299113216 | 0.021372615 |
| NDRG1 | Rigosertib | 0.297810125 | 0.021973332 |
| NDRG1 | LOXO-195 | 0.29647572 | 0.02260324 |
| NDRG1 | SCH-900776 | 0.29581509 | 0.022920702 |
| NDRG1 | IPI-145 | 0.295814542 | 0.022920967 |
| NDRG1 | CH-7057288 | 0.291664331 | 0.02500278 |
| NDRG1 | ENMD-2076 | 0.291535295 | 0.02506998 |
| NDRG1 | CC-115 | 0.289673838 | 0.02605645 |
| NDRG1 | 7-Hydroxystaurosporine | 0.2895972 | 0.026097754 |
| NDRG1 | Afuresertib | 0.289001931 | 0.026420455 |
| NDRG1 | AT-13148 | 0.288223401 | 0.02684756 |
| NDRG1 | PD 173074 | 0.286776971 | 0.027656488 |
| NDRG1 | GS-9901 | 0.284347069 | 0.029061463 |
| NDRG1 | SAR-125844 | 0.283898019 | 0.029327541 |
| NDRG1 | CCT-245737 | 0.28052666 | 0.031391069 |
| NDRG1 | Zoledronate | 0.279690575 | 0.031921166 |
| NDRG1 | RG-6016 | 0.278104802 | 0.032947106 |
| NDRG1 | LY-2090314 | 0.278097099 | 0.032952156 |
| NDRG1 | RX-5902 | 0.274483058 | 0.035393597 |
| NDRG1 | Rabusertib | 0.272170174 | 0.037033507 |
| NDRG1 | Cediranib | 0.270618246 | 0.038168795 |
| NDRG1 | KX-01 | 0.270167 | 0.03850425 |
| NDRG1 | GSK-690693 | 0.270057114 | 0.038586307 |
| NDRG1 | Staurosporine | 0.269901479 | 0.038702775 |
| NDRG1 | AMG-458 | 0.268237757 | 0.039966065 |
| NDRG1 | Seliciclib | 0.267040456 | 0.040896106 |
| NDRG1 | Telatinib | 0.266109106 | 0.041631842 |
| NDRG1 | LY-2801653 | 0.265461288 | 0.042150002 |
| NDRG1 | GDC-0349 | 0.263709012 | 0.043578232 |
| NDRG1 | MK-2206 | 0.259333506 | 0.047318958 |
| NDRG1 | relacorilant | 0.258422354 | 0.048130075 |
| NDRG1 | BMS-777607 | 0.257767088 | 0.0487204 |
| NDRG1 | BAY-1163877 | 0.256841675 | 0.049564164 |
| NDRG1 | Imatinib | -0.261920929 | 0.045076421 |
| NDRG1 | MPC-3100 | -0.264533155 | 0.042901617 |
| NDRG1 | ABL-001 | -0.272917097 | 0.036497166 |
| NDRG1 | DIGOXIN | -0.283619337 | 0.029493695 |
| NDRG1 | Dexrazoxane | -0.287492829 | 0.027253618 |
| NDRG1 | B-7100 | -0.30020408 | 0.020880492 |
| NDRG1 | By-Product of CUDC-305 | -0.301488373 | 0.02031346 |
| NDRG1 | AT-13387 | -0.307963261 | 0.017649787 |
| NDRG1 | tic10 | -0.34242287 | 0.007937857 |
| NDRG1 | AMONAFIDE | -0.346647802 | 0.00715306 |
